# Polymorphism and Divergence in Two Willow Species, *Salix viminalis* L. and *Salix schwerinii* E. Wolf

**DOI:** 10.1534/g3.111.000539

**Published:** 2011-10-01

**Authors:** Sofia Berlin, Johan Fogelqvist, Martin Lascoux, Ulf Lagercrantz, Ann Christin Rönnberg-Wästljung

**Affiliations:** * Department of Plant Biology and Forest Genetics, Uppsala BioCenter, Swedish University of Agricultural Sciences, SE-750 07 Uppsala, Sweden; † Department of Plant Ecology and Evolution, Evolutionary Biology Centre, Uppsala University, SE-752 36 Uppsala, Sweden; ‡ Laboratory of Evolutionary Genomics, CAS-MPG Partner Institute for Computational Biology, Chinese Academy of Sciences, Shanghai, China

**Keywords:** *Salix*, gene flow, species divergence, nucleotide polymorphism, linkage disequilibrium

## Abstract

We investigated species divergence, present and past gene flow, levels of nucleotide polymorphism, and linkage disequilibrium in two willows from the plant genus *Salix*. *Salix* belongs together with *Populus* to the Salicaceae family; however, most population genetic studies of Salicaceae have been performed in *Populus*, the model genus in forest biology. Here we present a study on two closely related willow species *Salix viminalis* and *S. schwerinii*, in which we have resequenced 33 and 32 nuclear gene segments representing parts of 18 nuclear loci in 24 individuals for each species. We used coalescent simulations and estimated the split time to around 600,000 years ago and found that there is currently limited gene flow between the species. Mean intronic nucleotide diversity across gene segments was slightly higher in *S. schwerinii* (*π_i_* = 0.00849) than in *S. viminalis* (*π_i_* = 0.00655). Compared with other angiosperm trees, the two willows harbor intermediate levels of silent polymorphisms. The decay of linkage disequilibrium was slower in *S. viminalis* compared with *S. schwerinii*, and we speculate that this is due to different demographic histories as *S. viminalis* has been partly domesticated in Europe.

The species concept is fundamental in evolutionary biology, but accurate classification of organisms is still far from trivial. The recent developments of genetic tools that allow identification of genetic differences among organisms have greatly changed our view of how to accurately assess what determines a true species. For instance, based on assessment of genetic diversity among individuals, it has become evident that morphologically similar organisms may form distinct species. And sometimes the opposite holds true, as morphologically dissimilar organisms may in fact have small genetic differences and even be members of the same species.

Particularly in plants, species may appear phenotypically diverged while still experiencing high levels of gene flow and little genetic differentiation throughout most of the genome. Species from the genus *Salix*, or willows, have often been used to illustrate this phenomenon as they have a reputation of being a taxonomic nightmare due to the anticipated high frequency of hybrids. However, the actual frequency of natural hybridization in willows has seldom been investigated thoroughly. As a matter of fact, in one of the early attempts to classify willows, Wichura (1865) found hybrids to be generally rare, although they could be extremely frequent locally, leading Darwin (1865) to express his bewilderment at the “extreme frequency of hybrid willows.”

In the present study, we aim to investigate the species concept in two closely related willow species using multilocus polymorphism data and to contrast this to what is known about the morphology and general biology of the two species. Species in the *Salix* genus are trees, shrubs, or subshrubs that, together with the genus *Populus* (poplars), are members of the Salicaceae family. *Populus* is recognized as a model genus for genetic and genomic studies in angiosperm trees with many resources available, such as the genome sequence of *Populus trichocarpa* (Tuskan *et al.* 2006). There are more than 300 *Salix* species, and they are widespread in both the Northern and the Southern hemispheres, excluding Australasia and New Guinea. Many species display rapid growth and high biomass yields and are therefore used for short rotation biomass production (Karp *et al.* 2011). *S. viminalis* L. and *S. schwerinii* E. Wolf are dioecious willows that are phenotypically very similar. Both are multistemmed shrubs with long and slender leaves and are commonly found along streams and rivers and in other wet areas. As other *Salix* species, the sex-ratio is often female biased (Alström-Rapaport *et al.* 1997; Ueno *et al.* 2007). Both species can also easily reproduce clonally, although the extent of clonal reproduction is not well documented in these two species. In *S. sachalinensis*, for example, clonal propagation was less important than expected (Ueno *et al.* 2007). *S. viminalis* has a vast natural distribution ranging from Ireland and United Kingdom in the west to Siberia in the east (Figure 1). The exact boundaries of the natural range in Western Europe are uncertain due to extensive cultivation in the past. In Scandinavia, it was introduced in the 18^th^ century and has since then spread (Larsson and Bremer 1991). This was confirmed with allozyme markers, which also showed differences among rivers in pattern of isolation by distance (IBD), less disturbed rivers showing a greater pattern of IBD than rivers that had experienced a higher level of anthropogenic disturbances (Lascoux *et al.* 1996). *S. schwerinii* has a smaller and more eastern natural range (Figure 1), and although the two species in many regions come close to each other, they are apparently rarely found growing together (Skvortsov 1968).

**Figure 1:**
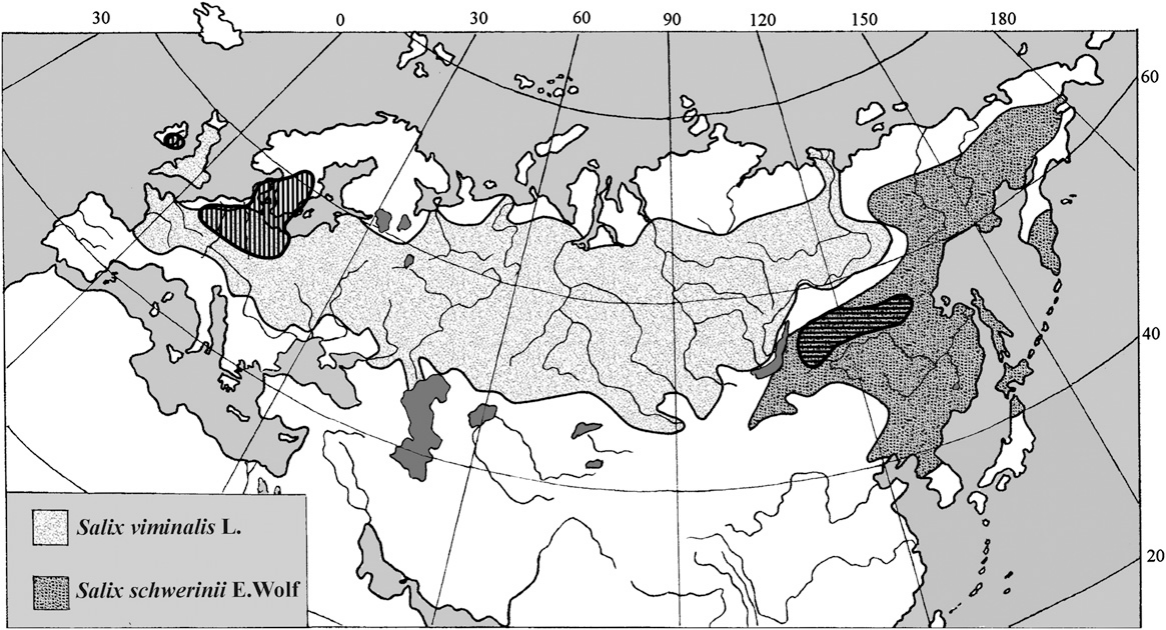
The natural ranges of *Salix viminalis* and *S. schwerinii*. Redrawn from Skvortsov (1968). The two dashed areas indicate the regions where samples were collected. Lakes are shown in dark gray color.

There are no reports of hybridization occurring in natural conditions, although they are easy to cross artificially in the laboratory. *S. viminalis* and *S. schwerinii* and their hybrids are some of the most commonly used *Salix* species in the breeding programs for biomass production in Europe. These species have also been the focus of most past investigations of *Salix* genetics, which include the generation of linkage maps (Berlin *et al.* 2010; Hanley *et al.* 2002; Hanley *et al.* 2007; Rönnberg-Wästljung *et al.* 2003; Tsarouhas *et al.* 2002) and QTL analyses (Rönnberg-Wästljung *et al.* 2005; Tsarouhas *et al.* 2003; Tsarouhas *et al.* 2004; Weih *et al.* 2006). In contrast to *S. viminalis*, *S. schwerinii* has not been used as extensively by humans and grows today in a geographical area in which the impact of the last glaciations was more limited. We would therefore expect the two species to show different patterns of nucleotide diversity and LD (Brubaker *et al.* 2005).

Level and pattern of nucleotide polymorphism and linkage disequilibrium (LD) as well as degree of population differentiation contain information about evolutionary forces that acted in the past and can be used to infer past demographic history. LD is determined by factors such as recombination, mutation, selection, and population admixture, as well as demographic history (Kim *et al.* 2007; Mueller 2004; Pritchard and Przeworski 2001). A population that has had a stable and large population size for a long time or has experienced a rapid size expansion will have lower levels of LD than small populations whose population size fluctuated through time or experienced recent bottlenecks (Mueller 2004; Pritchard and Przeworski 2001; Reich *et al.* 2001). The last scenario can be a consequence of domestication.

The aim of the present study is to investigate the species divergence between two phenotypically similar willow species, *S. viminalis* and *S. schwerinii*, with adjacent but presumably nonoverlapping natural ranges. We look for evidence of past and present gene flow both within species and between species. Considering both is important as gene flow and population dynamics of individual species will influence the amount of gene exchange among species (Petit and Excoffier 2009). We resequenced 33 and 32 nuclear loci that represent parts of 18 nuclear loci in the two species, and we determined levels and patterns of nucleotide diversity, population structure, and extent of linkage disequilibrium. We also used coalescent simulations to reconstruct demographic histories within each species and to estimate the degree of sequence divergence between species. More specifically, we asked whether polymorphisms and patterns of LD differ between the two species according to differences in biology.

## Materials and Methods

### Sampling

Twenty-four *S. viminalis* clones were included in the study. They originate from various localities in Europe and were selected to cover the western European part of the species range (Figure 1 and supporting information, Table S1). The clones were collected between 1978 and 1990 and are now growing in a field archive in Pustnäs, south of Uppsala. For details of the sampling, see Lascoux *et al.* (1996).

Twenty-four *S. schwerinii* clones were used, of which 17 were F1 crosses produced from different parental clones that are now also growing in the Pustnäs field archive. The six other clones are growing in a field archive in Svalöv, southern Sweden, and were kindly provided by Dr. Inger Åhman. The parents of the F1 crosses as well as the additional clones originate from Siberia, east of the Lake Baikal, and were collected in 1990 (Figure 1 and Table S1). Young leaves were sampled and stored in −20° awaiting DNA extraction. Genomic DNA was extracted with the FastDNA Kit (MP Biomedicals) according to the protocol provided with the kit, and DNA concentrations were determined with a Nanodrop spectrophotometer (Nanodrop Technologies).

### PCRs and DNA resequencing

We selected 20 protein coding genes from the *Populus trichocarpa* genome (http://www.phytozome.net/poplar.php) for resequencing in the willows, and for each gene, we attempted to resequence two segments (A and B) of approximately 500–1200 bp with various distances between them (Table 1). This method is known as the locus pair approach and has previously been used to study genetic diversity and linkage disequilibrium in humans (Frisse *et al.* 2001; Voight *et al.* 2005). This sampling scheme was used, as it will allow estimation of local as well as more long-range linkage disequilibrium without requiring extensive resequencing efforts. The poplar genome sequence served as template for the construction of PCR primers, and Primer3Plus was used for the primer design (Untergasser *et al.* 2007). The primers were positioned in exonic regions. Primer sequences that resulted in successful amplification and the positions of these primers in the poplar genome are provided in Table S2. The PCRs contained 10 ng genomic DNA, 1× PCR buffer II (Applied Biosystems), 2.5 mM MgCl_2_ (Applied Biosystems), 0.2 mM dNTP mix (Fermentas), 0.5 µM of each primer (Invitrogen), and 0.5 U AmpliTaq Gold DNA polymerase (Applied Biosystems) in a total of 15 μl. The reactions were run on a MyCycler thermal cycler (Bio-Rad Laboratories) with a PCR profile consisting of 10 min denaturation at 95° followed by 35 cycles of 30 s denaturation at 95°, 30 s annealing at 55°, and 1 min extension at 72° with a final 10 min 72° step. Amplification success was determined by agarose gel electrophoresis. The PCR products were cleaned with 1 μl of a mixture of Exonuclease I (New England BioLabs) and Shrimp Alkaline Phosphatase (SAP) (Fermentas) for every 5 μl of PCR product before they were directly sequenced at Macrogen, Inc., Europe (Macrogen, Amsterdam, Netherlands) using the forward and reverse PCR primers as sequencing primers. Sequences were edited, and contigs were assembled separately for each segment and sample using Seqman version 8.1.4 (Lasergene, DNASTAR), and heterozygous sites were scored with ambiguity codes. All sequences were carefully checked by eye. Two sequences were created for each sample in DAMBE version 5.2.5. (Xia and Xie 2001). As the poplar genome harbors many paralogs, BLAST searches against the genome database on the NCBI web site were performed with each of the gene segment to confirm that the orthologs had been amplified. All sequences are available in the NCBI Genbank sequence database (http://www.ncbi.nlm.nih.gov/genbank/) under accession numbers HQ625673–HQ628624.

**Table 1:** Location and size in base pairs (bp) of the amplified genes and gene segments

Gene	Segment	Position of segment in the poplar genome	Gene description*^b^*	Length (bp) of segment in poplar	Length (bp) of gene in poplar (length in willow)
I-1	I-1A	I: 274843–275686	Elongation factor P (EF-P) family protein	844	2911 (≈3000)
	I-1B	I: 272529–273628		1100	
I-53	I-53B*^a^*	I: 17978314–17978984	ACT domain-containing protein	671	
II-33	II-33A	II: 6600454–6601124	Putative tRNA modification GTPase	671	1918 (≈2000)
	II-33B	II: 6602004–6602412		409	
II-36	II-36A	II: 15840554–15841172	Arginyl-tRNA synthetase, class Ic	619	4602 (≈4000)
	II-36B	II: 15836568–15837300		733	
III-4	III-4A	III: 3483073–3483708	Lipase class 3 family protein	636	2904 (≈2800)
	III-4B	III: 3480736–3481702		967	
III-24	III-24A	III: 10971383–10971944	HSP70-interacting protein 1	562	3447 (≈3000)
	III-24B	III: 10968423–10969099		677	
IV-11	IV-11A	IV: 10288837–10289716	Ankyrin repeat family protein	880	3215 (≈3000)
	IV-11B	IV: 10291694–10292431		738	
IV-18	IV-18A	IV: 2906034–2906643	Putative peroxisomal (S)-2-hydroxy-acid oxidase 2	610	3266 (≈3500)
	IV-18B	IV: 2903319–2904194		876	
V-18	V-18A	V: 5944111–5944782	Formamidase	672	2918
	V-18B	V: 5941841–5942464		624	
V-20	V-20A	V: 14886605–14887432	Transcription factor jumonji (jmjC) domain-containing protein	828	1466
	V-20B	V: 14887398–14888114		717	
VI-4	VI-4A	VI: 3429957–3431097	Cytoplasmic tRNA 2-thiolation protein 2	1141	1769 (≈1800)
	VI-4B	VI: 3431048–3431789		742	
VII-1	VII-1A	VII: 179137–179680	Putative uridine nucleosidase 2	544	2723
	VII-1B	VII: 176958–177817		860	
VII-11	VII-11A	VII: 10593888–10594688	Transcription initiation factor TFIIB	801	1571 (≈1500)
	VII-11B	VII: 10593074–10593860		787	
VIII-5	VIII-5A	VIII: 4527231–4527936	Uncharacterized protein	706	5980
	VIII-5B	VIII: 4521896–4522960		1065	
VIII-14	VIII-14A	VIII: 13455553–13456551	Glucose-1-phosphate adenylyltransferase large subunit 1	999	3606 (≈4000)
	VIII-14B	VIII: 13459007–13459607		601	
VIII-22	VIII-22B*^a^*	Scaffold 132: 44890–44355	Anaphase-promoting complex subunit 7	607	
X-27	X-27A	X: 16861915–16862489	Transcription factor-like protein	575	6073
	X-27B	X: 16867064–16868053		990	
XII-8	XII-8A	XII: 7639702–7640377	Chromosome transmission fidelity protein 4	676	3144 (≈3000)
	XII-8B	XII: 7637193–7637994		801	

a Only one segment per gene.

b Best hit using BLASTX in the *Arabidopsis thaliana* genome.

### Confirmation of single nucleotide polymorphisms (SNPs) and of inferred haplotypes by cloning

Cloning of three segments I-53B, II-33B, and II-36A was performed using pGEM-T Easy Vector System I following the manufacturer’s protocols (Promega). PCR products of one to three *S. schwerinii* samples were cloned per gene segment. The PCRs were performed with 10 ng genomic DNA, 1× PCR buffer HF (Finnzyme), 0.2 mM dNTP mix (Fermentas), 0.3 µM of each primer (Invitrogen), and 0.2 U Phusion DNA polymerase (Finnzyme) in a total of 20 μl mixed and run on a MyCycler thermal cycler (Bio-Rad Laboratories) with a PCR profile consisting of 30 s at 98° followed by 30 cycles of 10 s at 98°, 30 s at 57°–60° and 2 min at 72° with a final step of 10 min at 72°. An A-nucleotide was added to the blunt-end PCR products prior to ligation according to the protocol found at (http://www.promega.com/pnotes/62/7807_15/7807_15_core.pdf). Ten colonies per sample were grown in overnight cultures, and plasmids were extracted using GeneJET Plasmid Miniprep Kit (Fermentas). Insert lengths were tested with PCR before sequencing with T7 and SP6 primers at Macrogen Inc. Cloned and directly sequenced DNA sequences were compared, and reconstructed haplotypes were compared with the cloned alleles.

### Sequence and SNP analysis

Sequence alignments were constructed for each segment and species using Clustal W (Thompson *et al.* 1994) in the Alignment Explorer tool of MEGA4 using default parameters (Tamura *et al.* 2007). Hardy-Weinberg equilibrium was tested for each SNP using Arlequin version 3.5.1.2 (Excoffier *et al.* 2005) for each species, and deviations from Hardy-Weinberg equilibrium were tested with exact tests using default settings.

### Nucleotide diversity

Calculations of standard population genetics parameters were carried out for each segment with the DnaSP software version 5.10 (Librado and Rozas 2009). The level of polymorphism for each segment was estimated both as haplotype and nucleotide diversities; exons and introns were analyzed separately. Indels were excluded from all analyses. The pairwise nucleotide diversity (π) (Nei 1987) and Watterson’s estimator (θ) (Watterson 1975) were obtained for intronic DNA (*π_i_*, *θ_i_*) and for synonymous (*π_s_*, *θ_s_*) and nonsynonymous sites (*π_a_*, *θ_a_*) separately. π and θ are expected to be almost the same for neutral sites at statistical equilibrium under mutation and genetic drift (Tajima 1989). Analyses of allele frequency spectra (Tajima's D) were done for each segment.

### Analyses of within-species population structure

The genetic structure within each species was tested using Structure version 2.3.3 (Pritchard *et al.* 2000). Sites that showed significant statistical association due to LD after Bonferroni correction (α = 0.05) were removed before the Structure analysis. To infer the structure of the sampled populations, the admixture model was used with a burn-in of 50,000 and a run length of 500,000 for a number of clusters from K = 1 to K = 7, allowing for correlation of allele frequencies between clusters. Ten independent runs per K were performed to ensure that the results were consistent. The most likely number of clusters was estimated with the original method from Pritchard *et al.* (2000) and with the ΔK statistics given in Evanno *et al.* (2005).

### Analyses of divergence between the species

The level of divergence between the species was investigated using Structure version 2.3.3 with similar criteria as above. The degree of genetic divergence between the two species, *F_ST_*, was assessed by a locus-by-locus analysis of molecular variance (AMOVA) in Arlequin version 3.5.1.2 (Excoffier *et al.* 2005). Significance was determined with 10,000 permutations. We also assessed the number of shared *vs.* fixed sites in DnaSP version 5.10 (Librado and Rozas 2009).

### Linkage disequilibrium and recombination

The diploid sequences were phased into haplotypes using PHASE version 2.1 with default parameters (Stephens and Donnelly 2003; Stephens *et al.* 2001). This was done for each segment independently. However, in V-20, VI-4, and VII-11, the A and B segments were located next to each other and primer pairs were overlapping; therefore, they were treated as single segments. The PHASE analysis in *S. viminalis* resulted in 97% of reconstructed haplotype pairs with > 90% posterior probability and 88% of reconstructed haplotype pairs with > 90% posterior probability in *S. schwerinii*. The levels of linkage disequilibrium between SNPs with frequencies > 0.05 in reconstructed haplotypes with posterior probabilities ≥ 0.90 was estimated as *r^2^*, the mean squared correlation in allelic state (Hill and Robertson 1968), using DnaSP version 5.10 (Librado and Rozas 2009). Significance of the associations between SNPs was determined with Fisher’s exact test with Bonferroni correction. The overall decay of LD was estimated by plotting *r^2^* against the physical distance between SNPs. We then fitted a nonlinear regression that yields a least-squares estimate of the average recombination parameter for all segments, ρ, given the empirical relationship between pairwise *r^2^* and physical distance between sites (Hill and Weir 1988).

To investigate the decay of LD over longer physical distances, we combined the A and B segments for each gene and again reconstructed haplotypes in PHASE. The results of the A and B segments combined were 93% haplotype pairs with 90% posterior probability in *S. viminalis* and 83% in *S. schwerinii*. LD was estimated as previously described, and a new dataset with *r^2^* was created for each species. These datasets contain *r^2^* from the combined A and B segments for each gene for which we estimated the complete segment lengths in willow (see below), as well as data based on the A and B segment separately in cases where we did not know the complete lengths or where only the A or B segment was sequenced for a specific gene. The first and the second dataset are not independent datasets, and in some instances, they contain the same data.

### Analysis of physical distances between segments in willow

With knowledge of the physical distance between the A and B segments in willow, we can study the decay of LD over larger physical distance. In *Populus trichocarpa*, the lengths varied among the genes and ranged from 1466 to 6073 base pairs (Table 1). To estimate the corresponding lengths in the willows, we used the forward primer in the first segment and reverse primer in the second segment in PCR. The lengths of the PCR products were determined by comparisons to a DNA size marker (MassRuler DNA ladder mix, Fermentas) on agarose gels. DNA from two *S schwerinii* and two *S. viminalis* clones were used and Phusion DNA polymerase was done according to the protocol previously described.

### Approximate Bayesian computation of individual species

The demographic history of each species and the time of the split between them were estimated using approximate Bayesian computation (ABC) as described in Blum and Francois (2010). First, a set of summary statistics was calculated for our dataset after individuals and sites with too many missing data were removed. Only the longest intron sequence for each investigated gene was used, resulting in 14 loci from 14 genes included in the analyses.

For each species separately, we tested three demographic models: a standard neutral model, a population growth model, and a bottleneck model. For each demographic model, prior distributions of the model parameters were constructed, and 10^6^ draws from these prior distributions were used to simulate coalescent genealogies using the coalescent simulator *ms* (Hudson 2002). The prior distributions were chosen as *U(min*, *max)* or *log-U(min*, *max)*, with *min* and *max* set at reasonable values following initial trials with different values of *min* and *max*. For each draw, 14 independent genealogies were simulated, with the number of sequences and base pairs per sequence corresponding to the 14 used loci. Summary statistics were calculated using *libsequence* (Thornton 2003). For summary statistics, we used the mean and variance of Watterson’s θ, Tajima’s D, and the number of haplotypes.

Next, the Euclidean distance (δ) between the simulated summary statistics and the observed summary statistics was computed and a proportion (*P_δ_*) of simulation outcomes with the smallest distance was retained. The value of *P_δ_* was set to 0.001. Initial studies indicated that the outcome of the analysis was rather consistent over a wide range of *P_δ_* values (0.001–0.1)), but the peaks in posterior estimates were sharpest at the lowest threshold of *P_δ_*.

Finally, the posterior distributions of parameters and the posterior model probabilities were estimated with the nonlinear regression approach [feed-forward neural network (FFNN) regression models] developed by Blum and Francois (2010) and implemented in the R package *abc*. This approach helps to overcome two problems inherent to the ABC approach, namely, the curse of dimensionality and model comparison (Blum and Francois 2010).

The neutral model simply consists of two parameters; the population mutation rate (θ = *4N_0_μ*) and the population recombination rate (*ρ = 4N_0_r*). The prior distributions of both parameters are log uniform distribution with min of 0.0001 and max of 1. The same priors for θ and ρ were used for the two other demographic models. The growth model has an additional parameter, the growth rate α. Its prior follows a log uniform distribution with min of 0.001 and max of 10. The bottleneck model assumes a constant population size *N_0_* until *t_b_* units of time backward in time when population size instantaneously drops to *N_b_* for *d* units of time after which the population size instantaneously increases to *N_0_*. All units of time are in *4N_0_* generations. The prior distributions of *t_b_* were uniform *U*(0, 10), and those of *d* were uniform *U*(0, 10). The bottleneck severity (*f = N_b_/N_0_*) was fixed at 0.02.

### Approximate Bayesian computation of species divergence

For the combined dataset of both species, we considered three models. First we tested a neutral population split model with migration, with parameters *N1* (size of population 1), *N2* (size of population 2), *t_s_* (time since populations diverged), *M = 4N_0_m* (symmetric migration rate between population 1 and population 2), and *N_anc_* (size of ancient population). The second model used was a population split model without migration defined as above but with *M* fixed at 0. Finally, we considered a model in which the two species did not split but interbred freely. To make this model as close as possible to the population split models, we also assumed a population size change to *N_anc_* at time of *t_s_*. The priors were initially set at a relatively large interval and run for 10^6^ iterations. Thereafter, the parameters were estimated, and new priors were constructed near the 99% credible interval of the estimated parameters. As summary statistics in the species divergence scenarios, we used the averages of Watterson’s θ, Tajima’s D, and the number of haplotypes in each population and in the combined data, as well as Wright’s *F_ST_* and the numbers of shared, fixed, and private polymorphisms.

### Model validation

To evaluate the fit of the models to the data, for each model, 10^4^ draws of the parameters were made from the estimated posterior distributions, and coalescent simulations were performed using those parameters. The simulations were summarized by the mean and variance of several summary statistics (see Table S3 and Table S4), and then compared with the observed data. For each summary statistic, the probability of observing a value lower than the observed value given the model was estimated. To identify outlier loci, this was also done for each segment separately. The overall fit of the data to the model and comparisons among models can be visualized using principal component analysis (PCA). The multilocus average and variance of summary statistics were used to compute the first two principal components (PC) of the data for each model. The summary statistics used were the same as provided in Table S3. The 95% confidence intervals (C.I.) of the two first components were plotted together with the predicted values of the data using the PCA as predictor.

## Results

### PCRs and resequencing

We designed 40 primer pairs to amplify two regions of 20 nuclear genes. Sequences of sufficient quality were obtained for at least one segment from 18 loci (Table 1). Sequences for I-53A, VIII-22A, XII-18A, and XII-18B were not of sufficient quality, and they were therefore not used in any analysis. The same applied to IV-18B and VII-1B in *S. schwerinii*. As a result, 32 loci were analyzed for sequence variation in *S. schwerinii* and 33 loci in *S. viminalis*, representing 18 genes located on 10 different linkage groups.

### Confirmation of SNPs and inferred haplotypes by cloning

A total of 2193 base pairs of cloned and directly sequenced PCR products were compared, and 15 SNPs were confirmed. In all cases, the DNA sequences of the cloned segments were identical to the DNA sequences obtained with direct sequencing. We confirmed that a reconstructed haplotype with posterior probability 1.0 was indeed correct. However, we also found that when the most probable haplotype pair has a probability between 0.3 and 0.7, the phase is likely to be incorrect.

### Hardy-Weinberg equilibrium and inbreeding coefficient

#### S. schwerinii:

Eleven percent of all SNPs deviated significantly from Hardy-Weinberg expectations (*P* ≤ 0.01), and in all cases, this was due to a deficit of heterozygote genotypes. The majority of the significant SNPs were located in three gene segments: II-36B, IV-18A, and VIII-5B (Table 2).

**Table 2:** Intronic nucleotide variation, haplotype diversity, and neutrality test in *S. schwerinii*

	Intronic nucleotide diversity					Haplotype diversity		Neutrality test Tajima’s D
Segment	N	L	S (singleton)	*θ_i_* (×10^−3^)	*π_i_* (×10^−3^)	N_h_	H_e_ (SD)	
I-1A	48	711	13 (3)	4.12	6.68	15	0.794 (0.043)	1.87
I-1B*^a^*	42	413	4 (1)	2.25	2.81	5	0.602 (0.041)	0.71
I-53B	42	332	7 (1)	4.90	5.57	11	0.886 (0.020)	0.37
II-33A	48	421	13 (3)	6.96	9.47	10	0.762 (0.036)	1.09
II-33B	42	156	6 (0)	12.69	8.94	8	0.672 (0.062)	1.11
II-36A	42	162	10 (1)	14.35	23.54	13	0.895 (0.022)	1.12
II-36B*^a^*	26	363	24 (1)	17.33	23.74	6	0.774 (0.048)	1.35
III-4B	28	764	12 (6)	4.04	2.91	12	0.860 (0.045)	−0.92
III-24A	44	305	14 (2)	10.55	12.69	15	0.848 (0.037)	0.63
III-24B*^a^*	36	167	5 (0)	7.22	12.93	5	0.629 (0.053)	2.06*
IV-11A	46	615	9 (1)	3.33	4.30	10	0.752 (0.041)	0.83
IV-18A	36	383	19 (0)	11.96	17.72	10	0.851 (0.041)	1.61
V-18A	48	304	5 (1)	3.71	3.61	7	0.746 (0.035)	−0.06
V-18B	48	436	6 (0)	3.10	3.94	8	0.754 (0.035)	0.67
V-20A	44	604	24 (7)	9.13	7.50	19	0.885 (0.032)	−0.60
V-20B*^a^*	48	146	4 (0)	6.17	10.48	4	0.579 (0.050)	1.60
VI-4A	42	224	6 (0)	6.22	10.16	5	0.630 (0.049)	1.67
VI-4B*^a^*	44	409	19 (7)	10.68	10.95	10	0.716 (0.057)	0.08
VII-1A	44	382	12 (4)	7.22	5.73	16	0.832 (0.050)	−0.63
VII-11A	42	615	25 (4)	9.45	11.31	23	0.952 (0.016)	0.66
VII-11B	40	499	13 (3)	6.12	3.04	10	0.708 (0.058)	−1.57
VIII-5A	38	401	9 (1)	5.34	7.36	6	0.691 (0.046)	1.12
VIII-5B	36	569	20 (0)	8.48	5.62	6	0.770 (0.037)	−1.23
VIII-14A	44	661	8 (2)	2.78	2.87	11	0.844 (0.032)	0.09
VIII-14B*^a^*	46	336	7 (1)	4.74	4.00	11	0.688 (0.071)	−0.42
VIII-22B*^a^*	48	292	1 (0)	0.77	1.38	2	0.403 (0.060)	1.07
X-27A	44	223	8 (0)	8.25	15.22	7	0.836 (0.021)	2.36*
XII-8A*^a^*	48	371	6 (0)	3.64	3.35	7	0.735 (0.046)	−0.20
Total	––	11.264	309 (49)	––	––	272	––	––
Average	42	402	11.0 (2)	6.98	8.49	10	0.753 (0.042)	0.59

N, number of analyzed sequences; L, number of compared nucleotide sites; S, number of segregating sites; *θ_I_*, Watterson’s estimator of theta for intronic sites; *π_i_*, pairwise nucleotide diversity for intronic sites; N_h_, number of haplotypes; H_e_, haplotype diversity.

a Fragment contains more than one intron.

#### S. viminalis:

Thirteen percent of all SNPs deviated significantly from Hardy-Weinberg expectations (*P* ≤ 0.01), and again this was due to a deficit of heterozygote genotypes. The majority of the significant SNPs were located in three gene segments: II-36B, IV-18B, and VIII-5A (Table 3).

**Table 3:** Intronic nucleotide variation, haplotype diversity, and neutrality test in *S. viminalis*

	Intronic nucleotide diversity					Haplotype diversity		Neutrality test Tajima’s D
Segment	N	L	S (singleton)	*θ_i_* (×10^−3^)	*π_i_* (×10^−3^)	N_h_ (SD)	H_e_ (SD)	
I-1A	46	595	12 (3)	4.59	2.79	7	0.446 (0.088)	−1.17
I-1B*^a^*	48	402	5 (0)	2.80	1.85	3	0.265 (0.080)	−0.83
I-53B	48	332	4 (0)	2.71	5.12	5	0.750 (0.032)	2.03
II-33A	48	400	16 (0)	9.01	14.13	7	0.732 (0.056)	1.77
II-33B	48	174	4 (0)	5.18	7.28	5	0.735 (0.029)	0.93
II-36A	42	162	7 (1)	10.04	15.95	5	0.639 (0.048)	1.61
II-36B*^a^*	30	344	11 (0)	8.07	10.59	2	0.331 (0.089)	1.00
III-4B	42	710	10 (3)	3.27	1.93	7	0.574 (0.085)	−1.21
III-24A	48	352	6 (0)	3.84	6.54	6	0.583 (0.074)	1.80
III-24B	32	270	4 (0)	3.68	4.62	5	0.631 (0.073)	0.65
IV-11A	48	607	12 (1)	4.45	3.68	8	0.340 (0.088)	−0.52
IV-18A	48	413	17 (7)	9.28	8.88	10	0.801 (0.039)	−0.13
IV-18B*^a^*	38	533	27 (2)	12.06	19.83	7	0.802 (0.030)	2.22*
V-18A	42	304	6 (1)	4.59	4.08	6	0.557 (0.079)	−0.29
V-18B	42	436	7 (0)	3.73	6.78	14	0.836 (0.044)	1.67
V-20A	30	620	6 (1)	2.44	2.45	6	0.655 (0.073)	0.10
V-20B*^a^*	48	160	2 (1)	2.82	2.62	4	0.409 (0.076)	−0.79
VI-4A	42	872	12 (5)	3.20	2.35	12	0.833 (0.038)	−0.81
VI-4B*^a^*	44	436	14 (5)	7.38	5.42	7	0.515 (0.038)	−0.82
VII-1A	32	364	14 (1)	9.55	9.88	13	0.865 (0.049)	−0.32
VII-1B*^a^*	48	355	18 (6)	11.43	9.60	11	0.836 (0.042)	1.75
VII-11B	44	535	15 (5)	6.45	7.55	15	0.872 (0.031)	0.54
VIII-5A	42	326	6 (1)	4.28	4.90	9	0.733 (0.059)	0.38
VIII-5B	20	819	31 (8)	10.67	11.01	7	0.679 (0.102)	0.00
VIII-14A	36	507	2 (0)	0.95	0.96	3	0.298 (0.093)	0.03
VIII-14B*^a^*	42	373	2 (1)	1.25	0.89	3	0.324 (0.081)	−0.53
VIII-22B*^a^*	42	292	7 (1)	5.57	3.68	6	0.634 (0.062)	−0.93
X-27A	42	411	17 (1)	9.61	5.97	7	0.515 (0.089)	−1.22
XII-8A*^a^*	48	347	7 (1)	4.55	6.08	6	0.777 (0.032)	0.90
Total	––	13.058	316 (57)	––	––	217	––	––
Average	42	435	10.5 (2)	5.78	6.55	7	0.625 (0.061)	0.24

N, number of analyzed sequences; L, number of compared nucleotide sites; S, number of segregating sites; *θ_i_*. Watterson's estimator of theta for intronic sites; *π_i_*, pairwise nucleotide diversity for intronic sites; N_h_, number of haplotypes; H_e_, haplotype diversity.

a Fragment contains more than one intron.

To conclude, the majority of the SNPs in both species that deviated from deviated significantly from Hardy-Weinberg expectations were located in three genes: II-36, IV-18, and VIII-5.

### Gene lengths in willow

For 12 genes, the PCRs resulted in specific PCR products similar to the expected lengths in poplar (Table 1) and could be used in the analysis of LD between segments in genes.

### Linkage disequilibrium

#### S. schwerinii:

**Figure 2:**
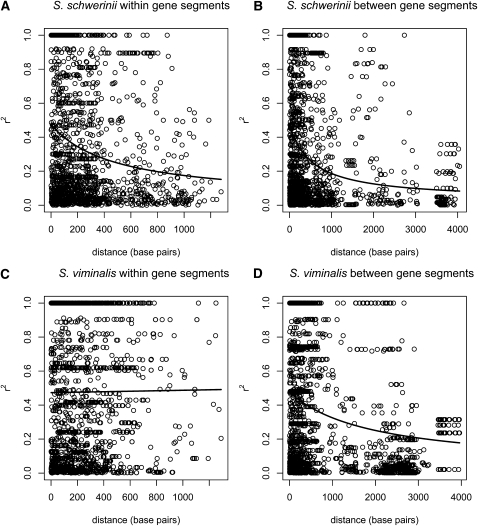
Patterns of linkage disequilibrium described as squared correlations between segregating sites: (A) *S. schwerinii* within gene segments, (B) *S. schwerinii* between gene segments, (C) *S. viminalis* within gene segments, and (D) *S. viminalis* between gene segments.

For each species, we have two datasets for which we estimated linkage disequilibrium (*r^2^*). In the first dataset, *r^2^* is estimated between SNPs located within segments, and in the second, *r^2^* is estimated between segments and within segments. Within the segments, average *r^2^* = 0.34, and among 1610 pairwise comparisons between SNPs, 436 were significant (*P* < 0.05) after Bonferroni correction (27%). LD decayed within the segments with *r^2^* dropping below 0.2 around 1000 base pairs (Figure 2A). The combined dataset allowed us to analyze the decay of LD between SNPs with a maximum distance of 4000 base pairs. The average *r^2^* = 0.31 and 350 out of 1862 pairwise comparisons (19%) were significant (*P* < 0.05) after Bonferroni correction. As in the above case, *r^2^* dropped below 0.2 around 1000 base pairs (Figure 2B).

#### S. viminalis:

We found more LD in *S. viminalis* than in *S. schwerinii*. Within the gene segments, average *r^2^* = 0.49, and among 1780 pairwise comparisons between SNPs, 591 were significant after Bonferroni correction (33%). LD did not decay between SNPs with maximum distance of 1300 base pairs (Figure 2C). As in *S. schwerinii*, with the combined dataset we could study LD between SNPs with up to 4000 base pairs distance, and we found that LD dropped below 0.2 after around 4000 base pairs (Figure 2D). The average *r^2^* = 0.40 and 484 out of 2476 pairwise comparisons (20%) were significant after Bonferroni correction.

### Nucleotide variation

#### S. schwerinii:

Intronic nucleotide variation was estimated in an average of 42 haplotypes, and a total of 11,264 base pairs from 28 loci were aligned (Table 2). We identified a total of 358 SNPs in the introns of which 49 were singletons. This corresponds to 1 SNP every 31 base pairs. Synonymous and nonsynonymous nucleotide variation was estimated in an average of 44 haplotypes and a total of 2188 base pairs from four loci (Table 4). We found a total of 28 synonymous SNPs of which 5 were singletons (1 SNP every 18 base pairs) and 22 nonsynonymous SNPs of which 3 were singletons (1 SNP every 77 base pairs). Five SNPs had 3 variants of which 3 were located in II-36A and one in III-24A and III-24B. Pairwise nucleotide diversity (*π_i_*) in introns was between 0.0014 and 0.0237 (average *π_i_* = 0.0085), and *θ_i_* ranged between 0.0008 and 0.0173 (average *θ_i_* = 0.0070). The synonymous pairwise nucleotide diversity (*π_s_*) was between 0.0036 and 0.0175 (average *π_s_* = 0.0095), and *θ_s_* ranged between 0.0057 and 0.0173 (average *θ_s_* = 0.0100). The nonsynonymous pairwise nucleotide diversity (*π_a_*) ranged between 0.0021 and 0.0041 (average *π_a_* = 0.0030), and *θ_a_* was between 0.0018 and 0.0031 (average *θ_a_* = 0.0025). The ratio between *π_a_*:*π_s_* = 0.278, and synonymous diversity was on average 26% greater than intronic nucleotide diversity. The mean Tajima’s D across loci was positive (0.59), but only two loci (III-24B and X-27A) showed significant positive Tajima’s D-values (Table 2).

**Table 4:** Synonymous and nonsynonymous nucleotide variation, haplotype diversity and neutrality test in *S. schwerinii*

		Synonymous sites				Nonsynonymous sites				Haplotype diversity		Neutrality test Tajima’s D, synonymous/nonsynonymous
Segment	N	L	S (singleton)	θ_s_ (×10^−3^)	π_s_ (×10^−3^)	L	S (singleton)	θ_a_ (×10^−3^)	π_a_ (×10^−3^)	N_h_ (SD)	H_e_ (SD)	
III-4A*^a^*	46	103	5 (1)	11.09	12.26	366	4 (1)	2.49	4.14	17	0.833 (0.050)	0.26/1.53
IV-11B*^a^*	40	79	2 (1)	5.95	3.57	260	2 (0)	1.81	2.53	5	0.632 (0.049)	−0.95/0.94
X-27B	42	162	12 (3)	17.26	17.47	519	7 (1)	3.13	3.07	19	0.923 (0.025)	0.04/−0.05
XII-8B	48	158	4 (0)	5.71	4.72	541	6 (1)	2.50	2.07	8	0.787 (0.042)	−0.40/−0.44
Total	—	502	23 (5)	—	—	1686	19 (3)	—	—	49	—	—
Average	44	126	5.8 (1)	10.00	9.51	422	4.8 (1)	2.48	2.95	12	0.794 (0.042)	−0.26/0.50

N, number of analyzed sequences; L, number of compared nucleotide sites; S, number of segregating sites; θ_s_ and θ_a_ is Watterson\s estimator of theta for synonymous sites and nonsynonymous sites; π_s_ and π_a_, pairwise nucleotide diversity for synonymous sites and nonsynonymous sites; N_h_, number of haplotypes; H_e_, haplotype diversity.

a Fragment contains more than one exon.

#### S. viminalis:

Intronic nucleotide variation was analyzed in an average of 42 haplotypes, and a total of 13,058 base pairs from 30 loci were aligned (Table 3). We identified a total of 373 SNPs in the introns of which 57 were singletons. This corresponds to 1 SNP every 35 base pairs. Synonymous and nonsynonymous nucleotide variation was estimated in an average of 47 haplotypes and a total of 2,475 base pairs from four loci (Table 5). We found a total of 10 synonymous SNPs of which 1 was a singleton (1 SNP every 56 base pairs) and 10 nonsynonymous SNPs of which none was a singleton (1 SNP every 191 base pairs). Two SNPs had 3 variants of which 1 was located in V-18B and 1 in V-20B. One SNP with 4 variants was found in VII-1A. Pairwise nucleotide diversity (*π_i_*) in introns was between 0.0009 and 0.0198 (average *π_i_* = 0.0066), and *θ_i_* ranged between 0.0010 and 0.0121 (average *θ_i_* = 0.0058). The synonymous pairwise nucleotide diversity (*π_s_*) was between 0 and 0.0217 (average *π_s_* = 0.0067), and *θ_s_* ranged between 0 and 0.0133 (average *θ_s_* = 0.0048). The nonsynonymous pairwise nucleotide diversity (*π_a_*) ranged between 0.0001 and 0.0039 (average *π_a_* = 0.0014), and *θ_a_* was between 0.00004 and 0.0038 (average *θ_a_* = 0.0013). The ratio between *π_a_*:*π_s_* = 0.238, and synonymous diversity was on average 7% greater than intronic nucleotide diversity (Table 5). As in *S. schwerinii*, the mean Tajima’s D across loci was positive (0.24), but only one segment (IV-18B) showed a significant positive Tajima’s D-value (Table 3).

**Table 5:** Synonymous and nonsynonymous nucleotide variation, haplotype diversity, and neutrality test in *S. viminalis*

		Synonymous sites				Nonsynonymous sites				Haplotype diversity		Neutrality test Tajima’s D, synonymous/nonsynonymous
Segment	N	L	S (singleton)	θ_s_(×10^−3^)	π_s_(×10^−3^)	L	S (singleton)	θ_a_(×10^−3^)	π_a_(×10^−3^)	N_h_(SD)	H_e_(SD)	
III-4A*^a^*	48	101	6 (1)	13.39	21.73	361	1 (0)	0.62	1.40	7	0.748 (0.037)	1.60/1.70
IV-11B*^a^*	42	110	2 (0)	4.22	4.53	367	6 (0)	3.80	3.87	8	0.727 (0.058)	0.14/0.04
X-27B	48	191	0 (0)	0.00	0.00	640	2 (0)	0.70	0.25	3	0.160 (0.070)	NA/−1.15
XII-8B	48	159	1 (0)	1.42	0.51	546	1 (0)	0.04	0.09	4	0.548 (0.032)	−0.87/1.70
Total	––	561	9 (1)	––	––	1914	10 (0)	––	––	22	––	––
Average	47	140	2	4.76	6.69	479	2.5 (0)	1.29	1.40	6	0.546 (0.049)	0.29/0.57

N, number of analyzed sequences; L, number of compared nucleotide sites; S, number of segregating sites; *θ_s_* and *θ_a_*, Watterson's estimator of theta for synonymous sites and nonsynonymous sites; *π_s_* and *π_a_*, pairwise nucleotide diversity for synonymous sites and nonsynonymous sites; N_h_, number of haplotypes; H_e_, haplotype diversity.

a Fragment contains more than one exon.

### Analyses of within-species population structure using Structure software

Based on 160 SNPs exhibiting no significant LD (*P* < 0.05, average *r^2^* = 0.024) from 24 *S. schwerinii* individuals, the most likely number of clusters was K = 3 both when the original method from Pritchard *et al.* (2000) was used and with the ΔK statistics given in Evanno *et al.* (2005) (data not shown). However, it is doubtful that there is any population structure in *S. schwerinii* because the difference in LnPD with K = 1, K = 2, and K = 3 is very small (average LnPD = −2450, −2459, and −2446, respectively). Further support is the clustering results for K = 3 (Figure S1A), which show no signs of structure for K = 3. Based on 80 SNPs exhibiting no significant LD (*P* < 0.05, average *r^2^* = 0.032) from 24 *S. viminalis* individuals, the most likely number of clusters using both methods was K = 2 with 6 and 18 individuals in the two groups (Figure S1B). Again, we believe that this structure has limited biological meaning as the differences in LnPD for the different K were rather small (average LnPD for K = 1–7 were −1299, −1200, −1207, −1273, −1286, −1301, and −1313, respectively).

### Analyses of divergence between the species

When analyzing both species using Structure, using 52 SNPs exhibiting no significant LD (*P* < 0.05, average *r^2^* = 0.047), both methods clearly supported K = 2 clusters, clearly separating the two species (Figure S1C and Figure S2). The locus-by-locus AMOVA analysis gave *F_ST_* = 0.56 (*P* < 0.0001). The total number of fixed differences was only 5, while the total number of shared polymorphisms was 71 (Table 6).

**Table 6:** The number of shared polymorphisms and fixed differences between *S. schwerinii* (1) and *S. viminalis* (2)

Segment	L	N1	N2	S1	S2	S, shared	S, fixed
I-1A	595	48	46	9	12	1	0
I-1B	379	42	48	4	5	1	0
I-53B	332	42	40	7	4	1	0
II-33A	399	48	48	13	16	8	0
II-33B	154	42	48	6	4	1	0
II-36A	162	42	42	10	7	5	0
II-36B	317	26	30	19	10	3	0
III-4A	462	46	48	8	7	3	0
III-4B	708	28	42	11	10	1	0
III-24A	305	44	48	14	4	3	0
III-24B	158	36	32	5	3	2	0
IV-11A	607	46	48	9	12	0	0
IV-11B	334	40	42	4	6	2	1
IV-18A	383	36	48	19	15	5	1
V-18A	304	48	42	6	10	1	0
V-18B	436	48	42	6	7	1	1
V-20A	603	44	30	24	5	1	0
V-20B	146	48	48	4	2	0	0
VI-4A	223	42	42	6	3	1	0
VI-4B	406	44	44	19	13	8	0
VII-1A	364	44	32	12	14	6	0
VII-11A	581	42	38	23	15	3	0
VII-11B	494	40	44	13	12	0	0
VIII-5A	196	38	42	3	4	0	0
VIII-5B	566	36	20	20	22	9	0
VIII-14A	475	44	36	5	2	0	1
VIII-14B	336	46	42	7	2	0	0
VIII-22B	292	48	42	1	7	0	1
X-27A	222	44	42	8	10	3	0
X-27B	685	42	48	19	2	1	0
XII-8A	347	48	48	6	7	0	0
XII-8B	700	48	48	10	2	1	0
Total	12671	––	––	330	254	71	5

L, number of compared nucleotide sites; N1, number of analyzed sequences in S. schwerinii; N2, number of analyzed sequences in *S. viminalis*; S1, number of segregating sites in *S. schwerinii*; S2, number of segregating sites in *S. viminalis*.

### ABC analysis of individual species

The observed data could best be explained by the standard neutral model in both species (*P* = 0.41 for *S. viminalis*, *P* = 0.47 for *S. schwerinii*), although the difference in probability between models was small, meaning that the alternative models could not be ruled out (Table 7). However, in the case of the growth model, the growth rate was very small (*α_mode_* = 0.0038 for *S. viminalis*, *α_mode_* = 0.0025 for *S. schwerinii*), and in the bottleneck model, a very ancient bottleneck was inferred (*tb_mode_* = 5.29 *N_e_* generations for *S.viminalis*, *tb_mode_* = 8.07 *N_e_* generations for *S. schwerinii*). Hence, in both cases, the posterior distributions of parameters suggest that a standard neutral model describes well the recent history of these two species. In this model, the population mutation rate θ was estimated to be 0.0026 (0.0014–0.0034) in *S. viminalis* and 0.0033 (0.0025–0.0044) in *S. schwerinii*, and the population recombination rate ρ was estimated to be 0.0036 (0.0007–0.0085) in *S. viminalis* and 0.0074 (0.0012–0.033) in *S. schwerinii*.

**Table 7:** Posterior estimates of model parameters in the per species models

Species	Model	θ	ρ	α	*t_b_*	*d*	*P*
*S. viminalis*	Neutral	0.0026 (0.0014–0.0034)	0.0036 (0.0007–0.0085)				0.41
	Growth	0.0027 (0.0019–0.0037)	0.00055 (0.0001–0.0090)	0.0038 (0.0009–0.51)			0.23
	Bottleneck	0.0031 (0.0022–0.0042)	0.0018 (0.0002–0.0088)		5.29 (1.02–9.39)	7.90 (−0.46–9.97)	0.36
*S. schwerinii*	Neutral	0.0033 (0.0025–0.0044)	0.0074 (0.0012–0.033)				0.47
	Growth	0.0040 (0.0026–0.0057)	0.021 (0.0057–0.041)	0.0025 (0.0013–0.37)			0.29
	Bottleneck	0.0027 (0.0016–0.0039)	0.0041 (0.0012–0.068)		8.07 (2.55–11.55)	4.61 (0.41–8.67)	0.25

Mode (95% C.I.).

θ, theta; ρ, rho; α, growth rate; *t_b_*, time since bottleneck end; *d*, duration of bottleneck; *P*, posterior model probability.

### ABC analysis of species divergence

**Figure 3:**
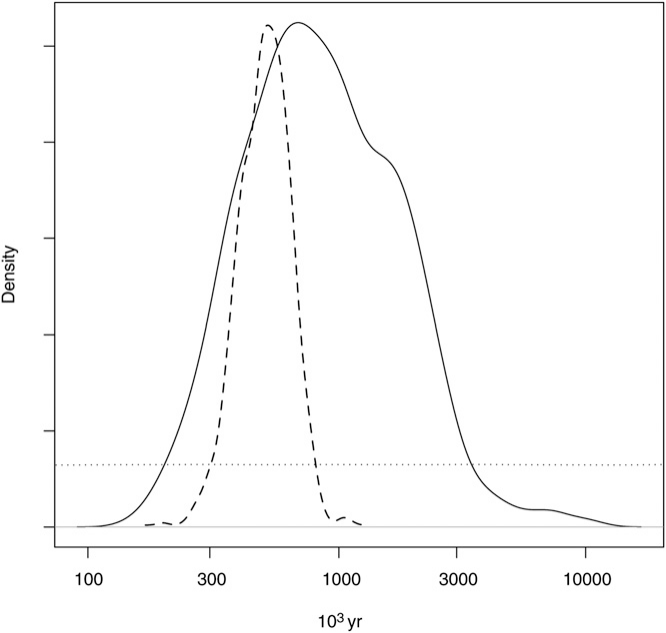
Posterior and prior distributions of population split time. Solid line: population split and migration; dashed line: population split without migration; dotted line: prior distribution. On the X-axis time in years since split event occurred assuming *N_e_* = 33 000 and a generation time of 2 years.

The observed data could best be explained using the population split model with or without migration and a rather recent split (*P_split_with_migration_* = 0.66, *P_split_no_migration_* = 0.31, *P_no_split_* = 0). The time since the population split event occurred was estimated to be 0.43 (0.16–1.98) × *4N_e_* generations with migration and 0.32 (0.19–0.47) × *4N_e_* generations without migration. Translating this to absolute time needs estimates of the effective population size, *N_e_*, and the mutation rate. Tuskan *et al.* (2006) estimated the synonymous substitution rate in the closely related genus *Populus* to be roughly 6-fold lower than in *Arabidopsis* to be compatible with fossil data. Using the Ossowski *et al.* (2010) estimate of μ = 5.9 × 10^−9^/year in *Arabidopsis*, this would translate to μ = 9.8 × 10^−10^/year in *Salix*, which is lower but of the same order of magnitude as the estimated mutation rate in Salicaceae in Berlin *et al.* (2010) (1.28 × 10^−9^ to 1.68 × 10^−9^). Assuming an approximate neutral mutation rate μ = 1 × 10^−9^/year, a generation time *g* of 10 years, and the posterior estimate of θ = 0.0016, *N_e_* would be *θ/4μg* = 40,000. Assuming this *N_e_*, the posterior estimate of split time in years would be 700,000 (260,000–3,100,000) with migration and 510,000 (300,000–752,000) without migration (Figure 3). Note that as we assume a mutation rate per year, the estimated split time in years will not depend on assumptions about generation time. While the relative size of the *S. viminalis* to the *S. schwerinii* population was close to 1 [0.80 (0.59–1.19)] with migration and 0.63 (0.36–1.04) without migration, the relative size of the ancient population to the current *S. viminalis* population size was estimated to be larger: 3.99 (0.59–11.26) with migration and 3.01 (1.44–6.06) without migration. The migration rate *M* was estimated to be 0.036 (0.002–0.11). θ was estimated to be 0.0016 (0.0008–0.0026) with migration and 0.0022 (0.0012–0.0037) without migration in line with the per species estimates. Also the ρ estimates were in line with the per species estimates: 0.017 (0.0089–0.028) with migration and 0.017 (0.0091–0.026) without migration. The model without a population split gave wide posterior estimates (Table 8).

**Table 8:** Posterior estimates of model parameters in the population split models

Model	θ	ρ	*N2*	*Nanc*	*M*	*ts*	*P*
Population split with migration	0.0016 (0.0008–0.0027)	0.017 (0.0089–0.028)	0.80 (0.51–1.19)	3.99 (0.59–11.26)	0.036 (0.002–0.11)	0.44 (0.16–1.98)	0.66
Population split without migration	0.0022 (0.0012–0.0037)	0.017 (0.0091–0.026)	0.63 (0.36–1.04)	3.01 (1.44–6.06)		0.32 (0.19–0.47)	0.35
No population split	0.0002 (0.0001–0.024)	0.0001 (<0.0001–0.0058)		0.16 (0.0041–22.90)		<0.0001 (0–6420)	0

Mode (95% C.I.).

θ, theta; ρ, rho; *N2*, relative size of *S. viminalis*; *Nanc*, relative size of ancestral population; *M*, migration; *ts*, time since population split between *S. schwerinii and S. viminalis* or since population size change in case of no population split model; *P*, posterior model probability.

### Validation of ABC models

**Figure 4:**
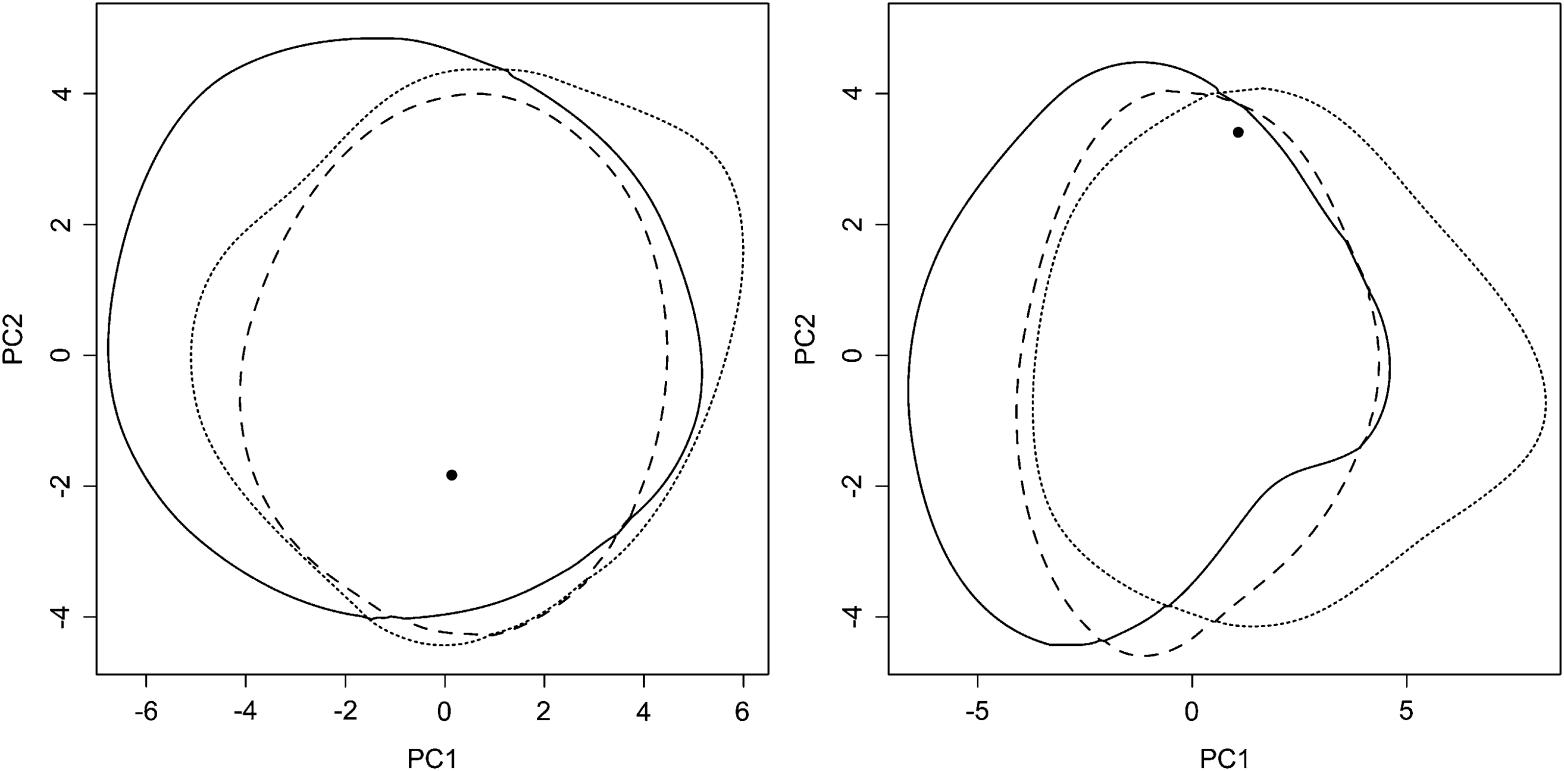
The two major principal component axes of the summary statistics obtained in the goodness of fit analysis of *S. viminalis* (left) and *S. schwerinii* (right) models. Within the plotted lines are 95% of the summary statistics. Solid line: bottleneck model; dotted line: growth model; dashed line: neutral model. The circle represents the fitted data.

**Figure 5:**
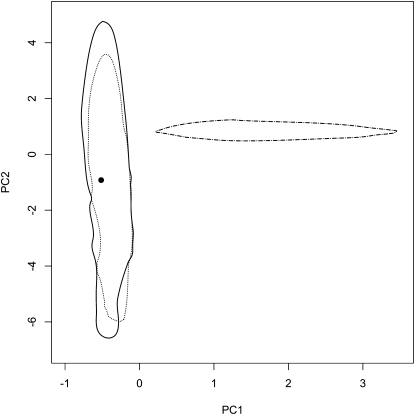
The two major principal component axes of the summary statistics obtained in the goodness-of-fit analysis of population split models. Within the plotted lines are 95% of the summary statistics. Solid line: population split with migration; dotted line: population split without migration; dash-dotted line: no population split. The circle represents the fitted data.

To assess the fit of data to ABC models, the data were summarized by the average and variance of several summary statistics, and then compared with the distributions of summary statistics obtained from coalescent simulations based on parameters drawn randomly from their posterior distributions. The differences between models in the analysis per species were low, hence, the fits of the models to the data were almost equal (Table S3, Table S4, Figure 4). Multilocus averages of neutrality test statistics (Tajima’s D, Fu and Li F* and D*) were higher in the data than expected under each model, although significantly different at α = 0.05 only for Tajima’s D and Fu and Li F* in *S. schwerinii*. The variance of Tajima’s D was also high in all models, but it was significantly higher than expected only in *S. viminalis* bottleneck and growth models. In the population split models, the difference between models was much more pronounced (Figure 5), at least when split and no split models were compared. In the population split model with migration, the same outlier loci were detected as in the per species models (Table S5 and Table S6). For the multilocus statistics, only the variance in number of haplotypes was significantly higher in the data (*P* = 0.98) in the population split with migration model, and no statistic at all in the population split without migration (Table S7). In the model without population split, the average and variance of *F_ST_* and number of fixed polymorphisms were significantly higher in the data compared with the model (*P* = 1) (Table S7).

## Discussion

### Species divergence

At present, both *S. viminalis* and *S. schwerinii* have widespread natural ranges with a possible contact zone in Eastern Russia. Even though the two species come close together in many regions, it seems that they are rarely growing together (Skvortsov 1968). Morphologically, *S. viminalis* is not entirely homogenous over its vast range; however, the observed differences are not considered enough to classify them as different taxonomic units (Skvortsov 1968), and overall, the two species are very similar in their vegetative appearance. Further support for the close relationship between *S. viminalis* and *S. schwerinii* is that they can easily be crossed in the laboratory. Initially, we were interested to know how genetically diverged the species are. Our resequencing effort shows that there are a large number of shared polymorphisms and very few fixed differences between the species. Such pattern could be the result either of secondary contact and recent gene flow or of incomplete lineage sorting since the split of the species. Our data support the last scenario because in species with large effective population sizes, a long time is required for alleles to go to fixation, even in the absence of gene flow (Hudson and Coyne 2002). Furthermore, our analyses show that most shared polymorphisms are ancestral or at least do not reflect recent gene flow, and the average split time was estimated to be on average 600,000 years ago. It is, however, difficult to detect gene flow occurring shortly after the two species separated (Becquet and Przeworski 2009), so we cannot rule out that some gene flow has occurred since the split. Further support of two clearly separated species come from a Structure analysis (Pritchard *et al.* 2000) with both species, where the most likely number of clusters was K = 2 and the two species clearly separated (Figure S1), as well as the high *F_ST_* value (0.56) estimated between them. To summarize the results so far, we have found that the two species diverged from a common ancestor rather recently and only restricted gene flow has occurred since then. Our data thus support limited hybridization between these species in nature. However, we cannot rule out the possibility that hybridization occurs in or near the contact zone as our samples do not originate from the area where the two species come in close contact. This is, for example, the case with *S. alba* L. and *S. fragilis* L. in central Europe that frequently hybridize in the contact zone to form *S*. × *rubens* Schrank (Kehl *et al.* 2008; Triest *et al.* 2009). However, outside the contact zone, hybridization does not seem to be substantial. We acknowledge that our sampling does not allow us to firmly conclude whether these two species are truly distinct or constitute a geographic gradient of one species. Field observations (Skvortsov 1968) support the view that they are truly distinct species, possibly adapted to different ecological niches because they are rarely found growing together. However, further genetic studies, including more extensive sampling throughout the natural range of each species, including the contact zone, are needed to settle this issue.

### Nucleotide diversity, LD, and demography

Mean intronic nucleotide diversity across gene segments was slightly higher in *S. schwerinii* (*π_i_* = 0.00849) than in *S. viminalis* (*π_i_* = 0.00655). These values are intermediate when compared with other angiosperm trees that demonstrate a great variation in synonymous and silent nucleotide diversities. Extreme values ranging from 0.012 to 0.016 have been observed in *Populus tremula* (Ingvarsson 2005; Ingvarsson 2008), and considerably lower values in *P. balsamifera* (0.0030–0.0045) (Keller *et al.* 2010; Olson *et al.* 2010), *P. tricocharpa* (0.0029–0.0035) (Gilchrist *et al.* 2006; Tuskan *et al.* 2006), *P. nigra* (0.0021), and *P. alba* (0.0035) (Giusi Zaina, personal communication, based on 31 fragments of *P. nigra*, 18 fragments of *P. alba*, and 12 genotypes in each species). Different effective population sizes have been put forward as an explanation for the difference in *π_s_* between *P. tremula* and *P. balsamifera* (Keller *et al.* 2010; Olson *et al.* 2010). This is supported by the difference in the ratio between *π_a_* and *π_s_* and the finding that nonsynonymous diversity is similar in the two species but the synonymous is much higher in *P. tremula* (Olson *et al.* 2010). Due to a smaller effective population size in *P. balsamifera*, purifying selection will be less efficient and deleterious nonsynonymous mutations will therefore accumulate, something that can be seen in the higher *π_a_*:*π_s_* ratio in *P. balsamifera* (0.267) compared with *P. tremula* (0.142) (Olson *et al.* 2010). In *S. viminalis* and *S. schwerinii*, *π_a_*:*π_s_* = 0.238 and 0.278 and are thus very similar to the values reported in *P. balsamifera*, suggesting that *P. tremula* could be an outlier in this respect. Because our data were limited, this would need to be confirmed with a larger dataset.

There were not many differences in nucleotide diversity between the species; however, linkage disequilibrium is more sensitive to capture recent departures from the standard neutral model, and indeed, there were greater differences in LD among the two *Salix* species compared with the differences in nucleotide diversities. LD was higher in *S. viminalis* (*r^2^* = 0.40–0.49) than in *S. schwerinii* (*r^2^* = 0.31–0.34), and LD decayed to 0.2 around 4000 base pairs in *S. viminalis* and around 1000 base pairs in *S. schwerinii*. The absolute values of these LD estimates should be treated with caution, however, as they are possibly inflated due to the way the haplotypic phase was determined, but as both species were affected in the same way, the comparison of relative values should remain valid. Difficulties in inferring the haplotype phase happened when one segment contained multiple heterozygotes, which were then excluded from further analysis. Similar to the situation for nucleotide diversities, there are great differences in LD among outcrossing angiosperm trees. In *P. tremula*, LD decayed rapidly to <0.05 in around a few hundred bases (Ingvarsson 2005); however, in *P. balsamifera*, values are more similar to those observed in *S. viminalis* (mean *r^2^* = 0.52), and LD did not decay in the gene fragments (Olson *et al.* 2010). These data are comparable as the phases were determined in a similar fashion. Many factors, such as recombination, mutation, selection, population admixture, and demographic history, affect LD, which could explain why LD differs so much between species. We can only speculate about the cause of the difference in LD between *S. viminalis* and *S. schwerinii*.

Because population structure may affect estimates of LD, we checked whether any of the species showed evidence for population subdivision. Each species was analyzed using Structure version 2.3.3 (Pritchard *et al.* 2000). The analyses revealed low levels of population subdivision in both species, which could certainly be a consequence of the limited sampling. However, it is also possible that these species do not demonstrate great population structures throughout their whole species ranges due to the high dispersal possibilities, as both seeds and pollen are partly wind dispersed. Even though the *Salix* genus is generally considered to be insect pollinated, several species are also known to be wind pollinated, as the flowers are morphologically intermediate between wind and insect pollination (Peeters and Totland 1999). So a possible cause for the difference in LD between the two species is different demographic histories. In contrast to *S. schwerinii*, *S. viminalis* has been used for basket making by humans for centuries, and in the eighteenth century, it was extensively used as a crop with several thousands of hectares of cultivated areas in, for example, France and Poland (refs. in Lascoux *et al.* 1996). It is thus possible that the longer LD in *S. viminalis* is an effect of bottlenecks at domestication events. To test this further, we performed coalescent simulations to find out whether any of the species deviated from the standard neutral model (SNM) using bottleneck and population growth models. Bottlenecks caused by founder or domestication events and population expansions triggered by advances and retreats of glaciers have resulted in deviations from the SNM in many natural plant populations (Siol *et al.* 2010). Although the majority of plant populations deviate from the SNM, our analyses show that neither *Salix* species do. This was particularly unexpected in *S. viminalis*. A likely explanation is that because demographic changes in *S. viminalis* occurred recently, they are not reflected in the site frequency spectrum or, at least, they were not captured by a dataset of the size used in the present study. This lack of deviation of the SNM in *Salix* is most likely again an effect of the large *N_e_* and long-distance dispersal.

## Conclusion

This is the first study to present data of species divergence and levels of nucleotide polymorphism and linkage disequilibrium in species from the genus *Salix*. The results suggest that *S. viminalis* and *S. schwerinii* split on average 600,000 years ago and that limited species-wide gene flow has occurred since the split. Both species harbor substantial amounts of genetic variation, which is likely due to large effective population sizes. The large effective population sizes are also possibly the reason why there are many shared polymorphisms and few fixed differences, despite the limited gene flow. Compared with other angiosperm tree species, the rate of decay of LD in both species is fairly slow, particularly in *S.viminalis*. The difference between the two *Salix* species is possibly due to different demographic histories: *S. viminalis* has been partly domesticated in Europe, whereas *S. schwerinii* has not. The rate of decay of LD has implications for the potential utility of association mapping as it effectively determines whether genome-wide scans are feasible or whether a candidate gene approach has to be used. The slow LD decay in these species suggests that haplotype structure may be strong and functional sites may be linked to nearby sites that have no influence on function. However, future studies are required to estimate LD across greater distances to determine the variation of LD across the genome.

## Supplementary Material

Supporting Information
