# Supplementary material for: Polymorphism and Divergence in Two Willow Species, Salix viminalis L. and Salix schwerinii E. Wolf
Source: G3 (Bethesda). 2011 Oct 1;1(5):387–400. doi: 10.1534/g3.111.000539 (PMC3276148; doi:10.1534/g3.111.000539)
Supplement: Supporting Information [file supp_1_5_387__index.html]

Supporting Information 

# Polymorphism and Divergence in Two Willow Species, *Salix viminalis* L. and *Salix schwerinii* E. Wolf

## Supporting Information for Berlin *et al.*, 2011

**Files in this Data Supplement:**

- Supporting Information - Figures S1 and S2 and Tables S1-S7 (PDF, 884 KB)
- Figure S1 - (a) Structure analysis of *S. schwerinii* when K = 3 clusters are assumed (b)Structure analysis of *S. viminalis* when K = 2 clusters are assumed (c)Structure analysis of *S. viminalis* and *S. schwerinii* when K = 2 clusters are assumed (PDF, 364 KB)
- Figure S2 - (a) Estimated number of clusters (K) obtained with Structure when *S. schwerinii* and *S. viminalis* were run together (b)Estimated number of clusters (K) obtained with Structure when *S. schwerinii* and *S. viminalis* were run together (PDF, 116 KB)
- Table S1 - Sample origins and clone names (PDF, 72KB)
- Table S2 - Primer sequences (PDF, 56 KB)
- Table S3 - P-values of obtaining multilocus summary statistic of the data under the individual species models (PDF, 68 KB)
- Table S4 - Number of outlier loci in the validation of the per species ABC models. Loci were considered outlier when two-sided P-value < 0.05 (PDF, 40 KB)
- Table S5 - Outlier loci in the validation of ABC models (PDF, 44 KB)
- Table S6 - Number of outlier loci in the validation of the population split ABC models (PDF, 44 KB)
- Table S7 - P-values of obtaining multilocus summary statistic of the data under three different models(PDF, 92 KB)
