# Supplementary material for: Polymorphism and Divergence in Two Willow Species, Salix viminalis L. and Salix schwerinii E. Wolf
Source: G3 (Bethesda). 2011 Oct 1;1(5):387–400. doi: 10.1534/g3.111.000539 (PMC3276148; doi:10.1534/g3.111.000539)
Supplement: Supporting Information [file supp_1.5.387_000539.pdf]

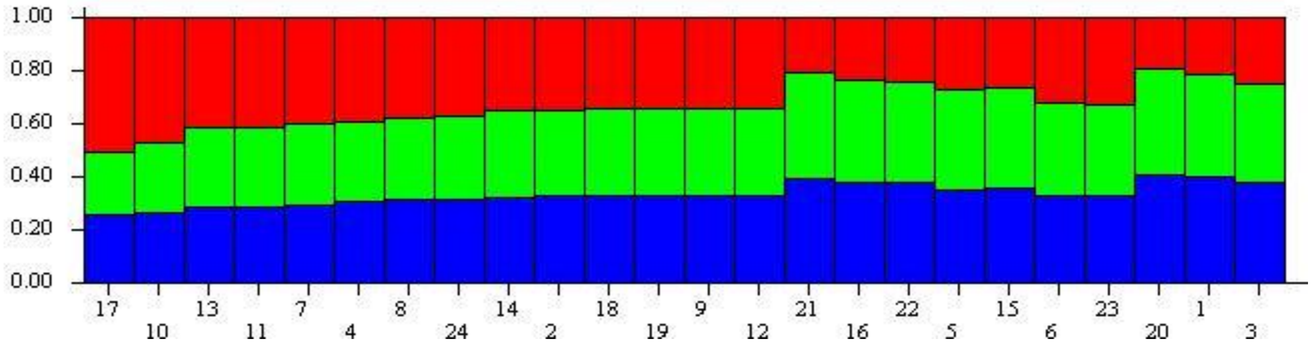

Figure S1a Structure analysis of *S. schwerinii* when K = 3 clusters are assumed.

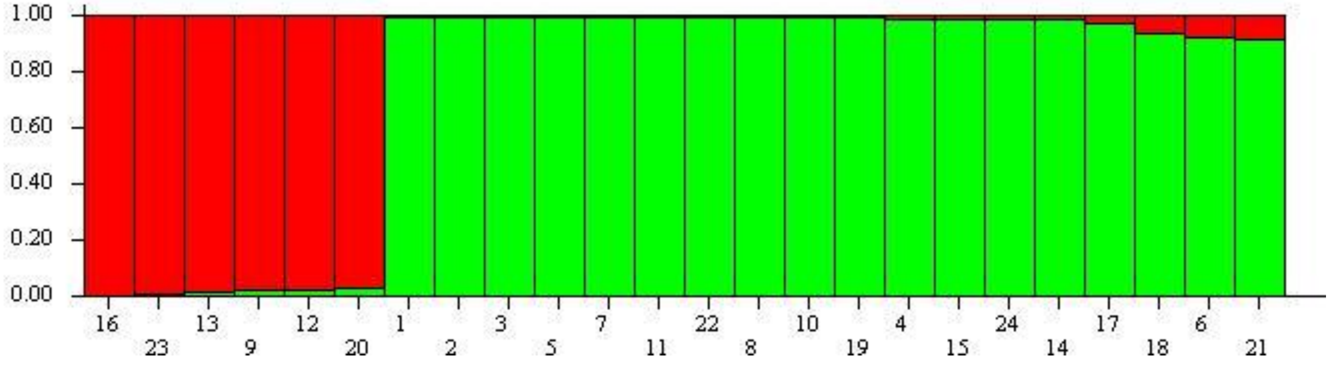

Figure S1b Structure analysis of *S. viminalis* when K = 2 clusters are assumed.

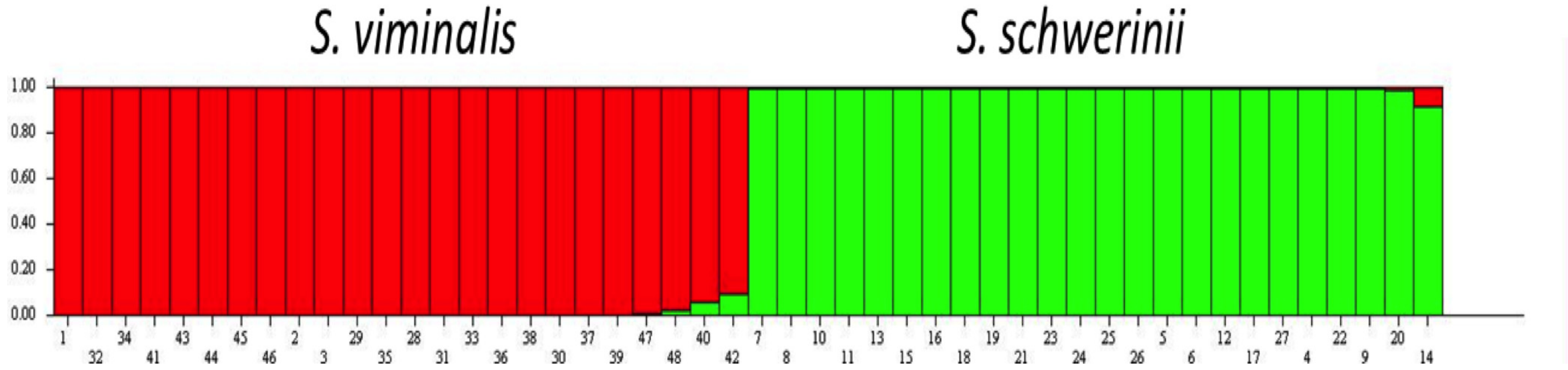

Figure S1c Structure analysis of *S. viminalis* and *S. schwerinii* when K = 2 clusters are assumed.

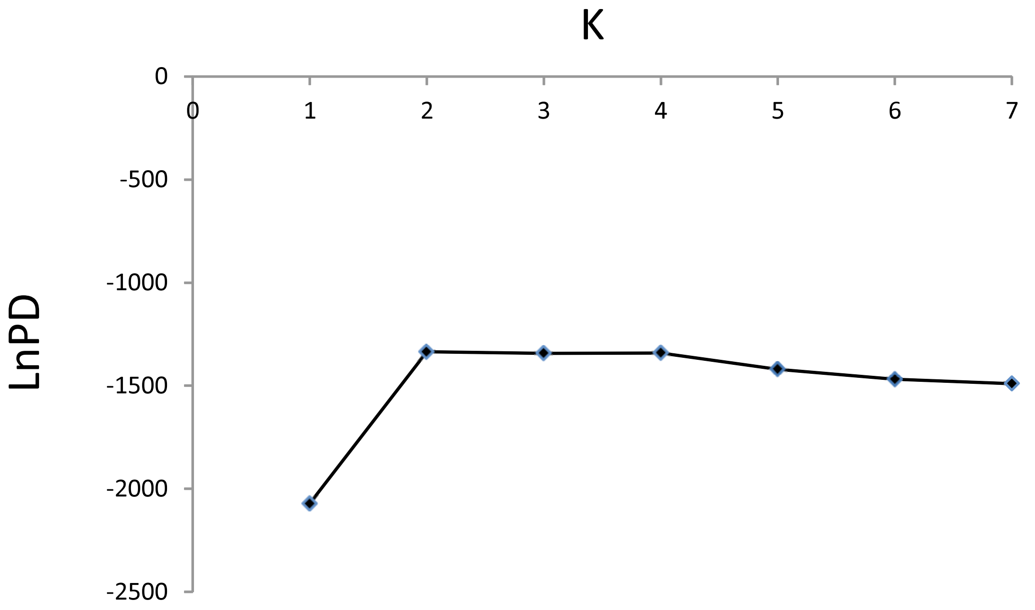

Figure S2a Estimated number of clusters (K) obtained with Structure when *S. schwerinii* and *S. viminalis* were run together. The mean LnPD is plotted over K (1-7).

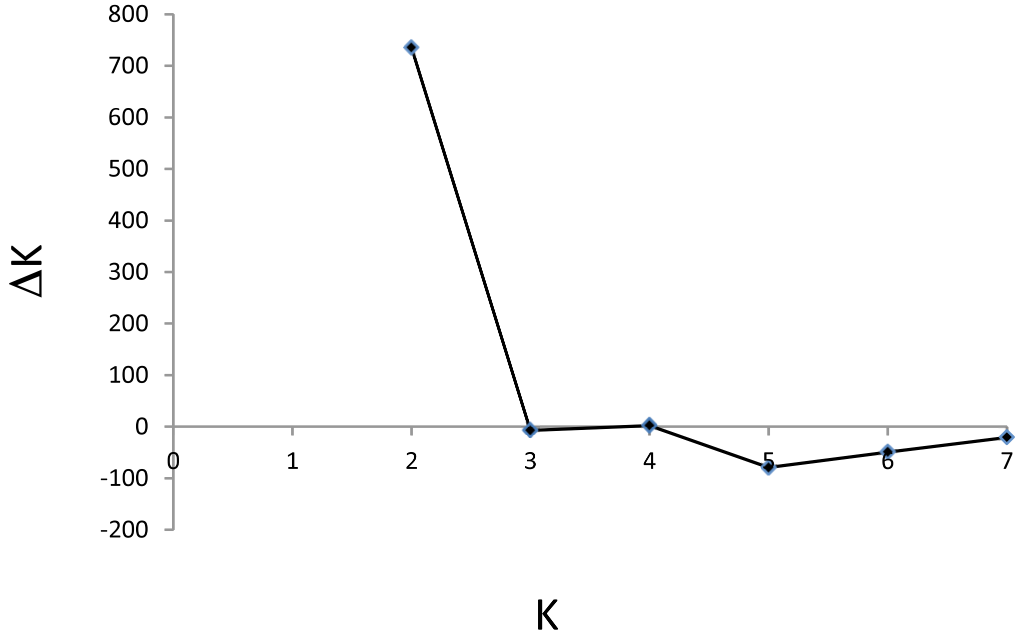

Figure S2b Estimated number of clusters (K) obtained with Structure when *S. schwerinii* and *S. viminalis* were run together. The mean ΔK is plotted over K (1-7).

Table S1 Sample origins and clone names

Species	Lab name	Sample name	Origin	Note
S. viminalis	V18	78183	Skåne, Sweden	
S. viminalis	V19	78195	Western Sweden	
S. viminalis	V52	81084	Southeast Sweden	
S. viminalis	V8	78054	Western Sweden	
S. viminalis	V14	78115	Southeast Sweden	
S. viminalis	V32	80019	North of malardalen Sweden	
S. viminalis	V40	80069	Denmark	
S. viminalis	V56	81102	Netherland	
S. viminalis	V59	82008	Skåne, Sweden	
S. viminalis	V69	82046	Ireland	
S. viminalis	V76	Bel34	Belgium	
S. viminalis	V85	Bel74	Belgium	
S. viminalis	V94	D161	Germany	
S. viminalis	V100	D252	Germany	
S. viminalis	V117	Linköp2A	Sweden	
S. viminalis	V119	P63	Poland	
S. viminalis	V121	P106	Poland	
S. viminalis	V126	P182	Poland	
S. viminalis	V136	skarp4	Sweden	
S. viminalis	V140	T19	Germany	
S. viminalis	V142	T76	Germany	
S. viminalis	V145	T114	Germany	
S. viminalis	V147	Öland7	Öland, Sweden	
S. viminalis	V208	90040	Sweden	
S. schwerinii	S1	96-1-40	parents: SW910260 + SW910259 collected at river Ingoda at the bridge Tchita	F1 cross
S. schwerinii	S2	96-2-18	parents: SW910261 + SW910267 collected at river Ingoda at the bridge Tchita	F1 cross

S. schwerinii	S3	96-5-6	parents: SW910282 + SW910284 collected at river Mongoi, village Mohov-Kondin	F1 cross
S. schwerinii	S4	96-6-15	parents: SW910287 + SW910285 collected at river Mongoi, village Mohov Kondin	F1 cross
S. schwerinii	S5	96-10-37	parents: SW910301+ SW910300 collected at river Vitim village Romanovka	F1 cross
S. schwerinii	S6	96-11-16	parents: SW910312 + SW910317 collected at the rivers Vitim & Bisjaga, village Bodaibo	F1 cross
S. schwerinii	S7	96-12-3	parents: SW910314 + SW910316 collected at the rivers Vitim & Bisjaga, village Bodaibo	F1 cross
S. schwerinii	S8	96-13-31	parents: SW910323 + SW910324 collected at the rivers Vitim & Bisjaga, village Bodaibo	F1 cross
S. schwerinii	S9	96-14-40	parents: SW910326 + SW910327 collected at river Tchitinka, village Tchita	F1 cross
S. schwerinii	S10	96-16-31	parents: SW910335 + SW910334 collected at river Tchitinka, village Tchita	F1 cross
S. schwerinii	S11	96-19-14	parents: SW910340 + SW910341 collected at rivers Shilka & Nertcha near to Nerchinsk	F1 cross
S. schwerinii	S12	96-20-23	parents: SW910343 + SW910344 collected at rivers Shilka & Nertcha near to Nerchinsk	F1 cross
S. schwerinii	S13	96-21-20	parents: SW910345 + SW910348 collected at rivers Shilka & Nertcha near to Nerchinsk	F1 cross
S. schwerinii	S14	96-22-24	parents: SW910349 + SW910350 collected at rivers Shilka & Nertcha near to Nerchinsk	F1 cross
S. schwerinii	S15	96-24-11	parents: SW910356 + SW910352 collected at rivers Shilka & Nertcha near to Nerchinsk	F1 cross
S. schwerinii	S16	96-25-31	parents: SW910368 + SW910369 collected at river Amur near Pokrova	F1 cross
S. schwerinii	S17	96-26-19	parents: SW910371+ SW910372 collected at river Amur near Pokrova	F1 cross
S. schwerinii	S18	SW911310	collected at Vitim o Bisjaga	
S. schwerinii	S19	SW911295	collected at river Vitim	
S. schwerinii	S20	SW911273	collected at river Mongoi	
S. schwerinii	S21	SW911329	collected at river Tchitinka	
S. schwerinii	S22	SW911283	collected at river Mongoi	
S. schwerinii	S23	SW911347	collected at rivers Shilka o Nertcha	
S. schwerinii	S24	SW911358	collected at river Amur	

Table S2   Primer sequences

Gene	Segment	Forward PCR primer 5´ to 3'	Reverse PCR primer 5´ to 3'
I-1	I-1A	TGATGGAAGACCTTTGTCTGG	GTCCAGCACAGCCTTTTTGT
	I-1B	CTTGGTTCGCAATCCAACACC	GGTTTGCTTGTTTCCACTGTC
I-53	I-53B*	CAGGTTTTGTGTGAGCTGGA	AGCAGGGCTCAGTGAATTGT
II-33	II-33A	AGGCTTTGCTTCTTTGGTCA	GCTGGACTTCCCAACATTAG
	II-33B	TAAGCATGTTTTCACCTGTGC	GCAGCATCCCTCAAATCAAT
II-36	II-36A	TGCTGTTTATTTGTTGTATGCAC	TCAGCAAATTGTAGCAAATGAA
	II-36B	TGAAGCAACAGTTCCAGGTG	GGGTGAGGAAAAGTCAACCA
III-4	III-4A	TTGTCTTCATGGTCGTGCAT	CAGCTTCCCATAAAGCGTTC
	III-4B	ACTGGAGCTGTTGTGCCTTT	CCACCAAGAGAGTGCCCTAC
III-24	III-24A	CCTGAATTGATGGCTGCATT	CACTTAGGCCCGGCAAATT
	III-24B	ATGATGGACGCAACAAAACT	TAGCCTTGGCCTCTTGAGAAG
IV-11	IV-11A	TGGTCACCACAAATTCGTCT	CATCTGCTGAGGACATGATTG
	IV-11B	CATGCCTCTCTCGGTTTCTC	AGTGCAAGCATGGGAAGTTG
IV-18	IV-18A	GTACAAGGCCGTGTTCCTGT	GCCATCGTCAGCTCAAACTC
	IV-18B	GAGTATCAGGAAATTGCAAGGC	CTGCTGAAGCTGCTCTAGCTG
V-18	V-18A	TGCTATCATGTTGCCCATGTG	TGGGCAAGTTTCCATCATAAG
	V-18B	GATTTCTGGAATAGTCGATTC	CAAGTTTCCATCATAAGTGC
V-20	V-20A	CCAAGGCTTTTATGGTGCAT	GAACTCCATCACCAAATTCCA
	V-20B	GCATGCAAGAAACAGGGTTTAC	AAGGATTGCATCCATTGCAG
VI-4	VI-4A	CAAGGATTGCTTGCCATGT	TGGCACAACAAATTCAGCTC
	VI-4B	GTGCTTCCACTTCGTGACTG	ATTGCAAATGGGACAGAACA
VII-1	VII-1A	GGCAGCAGAACCAAAGAAGA	TTCCTGGTGGCTAAAGTGGT
	VII-1B	CGATCCTGATGCTGCTGATA	GACAACAGTGGGAGCATCAA
VII-11	VII-11A	TTGGGAGAAGCTTTGCAACT	GGTGCAAAAGCATTTGTTGA
	VII-11B	ATTTTGGGCTTGGATTGTGA	TTGAGGTACATTGGCAGCAA
VIII-5	VIII-5A	TGGATTTCTGGACCAGGCTAC	GACAGGAGAATAGGAGCTTCG

	VIII-5B	TGCCATTCTACACCGAGTTG	AACCTGAAACCAGAGCACAG
VIII-14	VIII-14A	AAACTCCTGGAGAGGCAGG	CTGCACAAAGTCCATGTAGTC
	VIII-14B	TGCTTGGTGCTGACTTTTATG	ATGACCGTAACACCAGATCG
VIII-22	VIII-22B*	TCATGTCATTGAGGGTCGAA	TCCTGGTCTCCATCTGCATC
X-27	X-27A	TGTCAATGCCAAACCTTGAA	TTCAGTGGCTGGAACCTTCT
	X-27B	ACAGCCAAAACCAACACTCCG	ATCTCTCGTGGCACCTATGGA
XII-8	XII-8A	GGCATATTCTCTCCCTTGATG	GGGAGCCAGTTACCACCATA
	XII-8B	TGGTGACGATGAAGGCATTA	GGGTTCAGTCTTTGGAAGCA

*Only one segment per gene.

Table S3 P-values of obtaining multilocus summary statistic of the data under the individual species models. P-values are not corrected for multiple tests

Statistic	*S. schwerinii*			*S. viminalis*		
	Bottleneck	Growth	Neutral	Bottleneck	Growth	Neutral
Mean(S)	0,85	0,33	0,65	0,30	0,61	0,60
Mean(num_singleton)	0,46	0,08	0,20	0,08	0,13	0,13
Mean(πw)	0,76	0,23	0,47	0,24	0,48	0,46
Mean(TajD)	0,99	0,99	1	0,93	0,91	0,90
Mean(FuLiF*)	0,99	0,99	0,99	0,97	0,96	0,96
Mean(FuLiD*)	0,95	0,97	0,95	0,97	0,96	0,96
Mean(num_haplotypes)	0,76	0,23	0,70	0,30	0,58	0,55
Mean(DandVH)	0,68	0,16	0,52	0,11	0,21	0,15
Mean(WallsB)	0,61	0,71	0,50	0,94	0,91	0,91
Mean(WallsQ)	0,60	0,64	0,46	0,95	0,94	0,94
Var(S)	0,98	0,94	0,97	0,82	0,88	0,88
Var(num_singleton)	0,71	0,41	0,54	0,35	0,50	0,49
Var(πw)	0,60	0,25	0,38	0,32	0,46	0,44
Var(TajD)	0,88	0,96	0,88	0,98	0,98	0,97
Var(FuLiF*)	0,39	0,53	0,41	0,77	0,74	0,75

Var(FuLiD*)	0,13	0,20	0,14	0,66	0,64	0,66
Var(num_haplotypes)	0,92	0,92	0,95	0,91	0,90	0,90
Var(DandVH)	0,23	0,60	0,37	0,88	0,80	0,82
Var(WallsB)	0,50	0,76	0,48	0,69	0,57	0,55
Var(WallsQ)	0,46	0,70	0,42	0,60	0,48	0,45

TABLE S4 Number of outlier loci in the validation of the per species ABC models. Loci were considered outlier when two-sided P-value < 0.05. P values are not corrected for multiple tests.

Statistic		S. schwerinii			S. viminalis	
	Bottleneck	Growth	Neutral	Bottleneck	Growth	Neutral
Segregating sites	2	1	2	1	1	1
Singletons	0	0	0	0	0	0
Wattersons theta	2	1	2	1	1	1
Tajimas D	4	4	4	2	2	2
Fu & Li F*	3	3	3	2	2	2
Fu & Li D*	1	1	1	2	2	2
Number of haplotypes	1	1	1	2	2	3

Table S5 Outlier loci in the validation of ABC models. Number of statistics for which the loci were considered to be outlier at α=0.05 level, without correction for multiple tests.

Locus		*S. schwerinii*			*S. viminalis*				
	Bottleneck	Growth	Neutral	Bottleneck	Growth	Neutral	Split	Split without migration	No split
I-1	1	1	1	3	3	3	3	3	6
I-53b									3
II-33									3
II-36				2	2	2			4
III-4c							3	2	3
IV-11									3
IV-18	5	4	5					1	7
V-18b									2
VI-4	2	2	2	3	3	3			5
VII-11	3	3	3			1	3	1	2
VII-1				2	2	2			3
VIII-14								1	2
VIII-5									2
X-27	2	2	2						4

Table S6 Number of outlier loci in the validation of the population split ABC models. Loci were considered outlier when two-sided P-value < 0.05. P values are not corrected for multiple tests.

Statistic	Split	Split without migration	No split
Segregating sites $_{S.\ schwerinii}$			
Singletons $_{S.\ schwerinii}$			
Wattersons theta $_{S.\ schwerinii}$			
Tajimas D $_{S.\ schwerinii}$			4
Fu & Li F* $_{S.\ schwerinii}$			3
Fu & Li D* $_{S.\ schwerinii}$		1	1
Number of haplotypes $_{S.\ schwerinii}$	1	1	
Segregating sites $_{S.\ viminalis}$			
Singleton $_{S.\ viminalis}$			
Wattersons theta $_{S.\ viminalis}$			
Tajimas D $_{S.\ viminalis}$	1	1	4
Fu & Li F* $_{S.\ viminalis}$	1	1	3
Fu & Li D* $_{S.\ viminalis}$	1	1	2
Number of haplotypes $_{S.\ viminalis}$			
Segregating sites $_{S.\ schwerinii + S.\ viminalis}$			
Singletons $_{S.\ schwerinii + S.\ viminalis}$	2		
Wattersons theta $_{S.\ schwerinii + S.\ viminalis}$			
Tajimas D $_{S.\ schwerinii + S.\ viminalis}$	1	1	2
Fu & Li F* $_{S.\ schwerinii + S.\ viminalis}$	1	1	1
Fu& Li D* $_{S.\ schwerinii + S.\ viminalis}$			1
Number of haplotypes $_{S.\ schwerinii + S.\ viminalis}$	1		
Fst		1	14
Number of shared polymorphisms			
Number of fixed polymorphisms			14
Number of private polymorphisms $_{S.\ schwerinii}$			
Number of private polymorphisms $_{S.\ viminalis}$			

Table S7 P-values of obtaining multilocus summary statistic of the data under three different models. P values are not corrected for multiple tests.

Stat	Split	Split no migration	No split
Mean($S_{S.\ schwerinii}$)	0.72	0.43	0.87
Mean($singleton_{S.\ schwerinii}$)	0.71	0.47	0.86
Mean($\pi w_{S.\ schwerinii}$)	0.67	0.36	0.87
Mean($TajD_{S.\ schwerinii}$)	0.85	0.80	0.93
Mean($FuLiF^*_{S.\ schwerinii}$)	0.72	0.57	0.88
Mean($FuLiD^*_{S.\ schwerinii}$)	0.61	0.42	0.74
Mean($num\_haplo_{S.\ schwerinii}$)	0.72	0.40	0.89
Mean($DandVH_{S.\ schwerinii}$)	0.64	0.31	0.88
Mean($WallsB_{S.\ schwerinii}$)	0.38	0.23	0.52
Mean($WallsQ_{S.\ schwerinii}$)	0.39	0.22	0.54
Mean($S_{S.\ viminalis}$)	0.68	0.52	0.87
Mean($singleton_{S.\ viminalis}$)	0.63	0.57	0.85
Mean($\pi w_{S.\ viminalis}$)	0.65	0.48	0.86
Mean($TajD_{S.\ viminalis}$)	0.46	0.25	0.79
Mean($FuLiF^*_{S.\ viminalis}$)	0.59	0.38	0.80
Mean($FuLiD^*_{S.\ viminalis}$)	0.64	0.46	0.75
Mean($num\_haplo_{S.\ viminalis}$)	0.59	0.49	0.87
Mean($DandVH_{S.\ viminalis}$)	0.45	0.32	0.86
Mean($WallsB_{S.\ viminalis}$)	0.66	0.43	0.81
Mean($WallsQ_{S.\ viminalis}$)	0.69	0.45	0.85
Mean($S_{combined}$)	0.61	0.44	0.88
Mean($singleton_{combined}$)	0.73	0.53	0.87
Mean($\pi w_{combined}$)	0.55	0.35	0.88
Mean($TajD_{combined}$)	0.56	0.68	0.92
Mean($FuLiF^*_{combined}$)	0.52	0.56	0.89
Mean($FuLiD^*_{combined}$)	0.49	0.47	0.73
Mean($num\_haplo_{combined}$)	0.68	0.42	0.91
Mean($DandVH_{combined}$)	0.52	0.29	0.90

Mean($WallsB_{combined}$)	0.23	0.18	0.54
Mean($WallsQ_{combined}$)	0.24	0.19	0.55
Mean(Fst)	0.44	0.57	1.00
Mean(shared)	0.68	0.49	0.81
Mean(fixed)	0.30	0.26	1.00
Mean($private_{S.\ schwerinii}$)	0.76	0.46	0.90
Mean($private_{S.\ viminalis}$)	0.70	0.58	0.90
Var($S_{S.\ schwerinii}$)	0.86	0.78	0.89
Var($singleton_{S.\ schwerinii}$)	0.73	0.58	0.87
Var($\pi w_{S.\ schwerinii}$)	0.58	0.32	0.87
Var($TajD_{S.\ schwerinii}$)	0.77	0.86	0.81
Var($FuLiF*_{S.\ schwerinii}$)	0.44	0.57	0.52
Var($FuLiD*_{S.\ schwerinii}$)	0.24	0.35	0.46
Var($num\_haplo_{S.\ schwerinii}$)	0.97	0.93	0.91
Var($DandVH_{S.\ schwerinii}$)	0.26	0.52	0.45
Var($WallsB_{S.\ schwerinii}$)	0.34	0.40	0.69
Var($WallsQ_{S.\ schwerinii}$)	0.34	0.41	0.69
Var($S_{S.\ viminalis}$)	0.78	0.65	0.88
Var($singleton_{S.\ viminalis}$)	0.70	0.64	0.86
Var($\pi w_{S.\ viminalis}$)	0.60	0.42	0.86
Var($TajD_{S.\ viminalis}$)	0.91	0.87	0.95
Var($FuLiF*_{S.\ viminalis}$)	0.74	0.76	0.77
Var($FuLiD*_{S.\ viminalis}$)	0.71	0.76	0.66
Var($num\_haplo_{S.\ viminalis}$)	0.66	0.62	0.90
Var($DandVH_{S.\ viminalis}$)	0.54	0.65	0.82
Var($WallsB_{S.\ viminalis}$)	0.48	0.36	0.74
Var($WallsQ_{S.\ viminalis}$)	0.43	0.34	0.74
Var($S_{combined}$)	0.90	0.87	0.89
Var($singleton_{combined}$)	0.93	0.89	0.89
Var($\pi w_{combined}$)	0.39	0.23	0.87
Var($TajD_{combined}$)	0.85	0.91	0.72

Var($FuLiF^*_{combined}$)	0.85	0.88	0.62
Var($FuLiD^*_{combined}$)	0.71	0.74	0.52
Var($num\_haplo_{combined}$)	0.98	0.95	0.92
Var($DandVH_{combined}$)	0.60	0.73	0.42
Var($WallsB_{combined}$)	0.31	0.40	0.69
Var($WallsQ_{combined}$)	0.37	0.46	0.70
Var(Fst)	0.75	0.80	1.00
Var(shared)	0.76	0.61	0.86
Var(fixed)	0.28	0.24	1.00
Var($private_{\textit{S. schwerinii}}$)	0.89	0.81	0.90
Var($private_{\textit{S. viminalis}}$)	0.77	0.69	0.90
