# Supplementary material for: Polymorphism and Divergence in Two Willow Species, Salix viminalis L. and Salix schwerinii E. Wolf
Source: G3 (Bethesda). 2011 Oct 1;1(5):387–400. doi: 10.1534/g3.111.000539 (PMC3276148; doi:10.1534/g3.111.000539)
Supplement: Supporting Information [file supp_1.5.387_FigureS1.pdf]

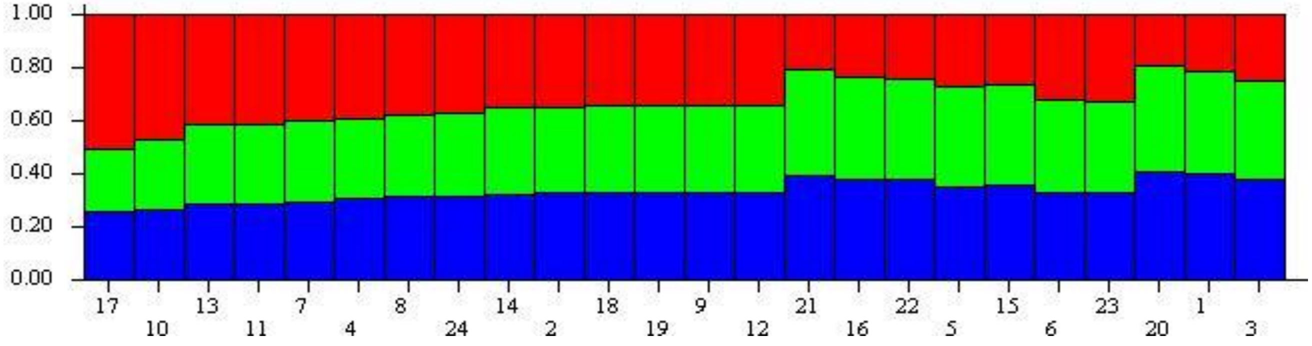

Figure S1a Structure analysis of *S. schwerinii* when K = 3 clusters are assumed.

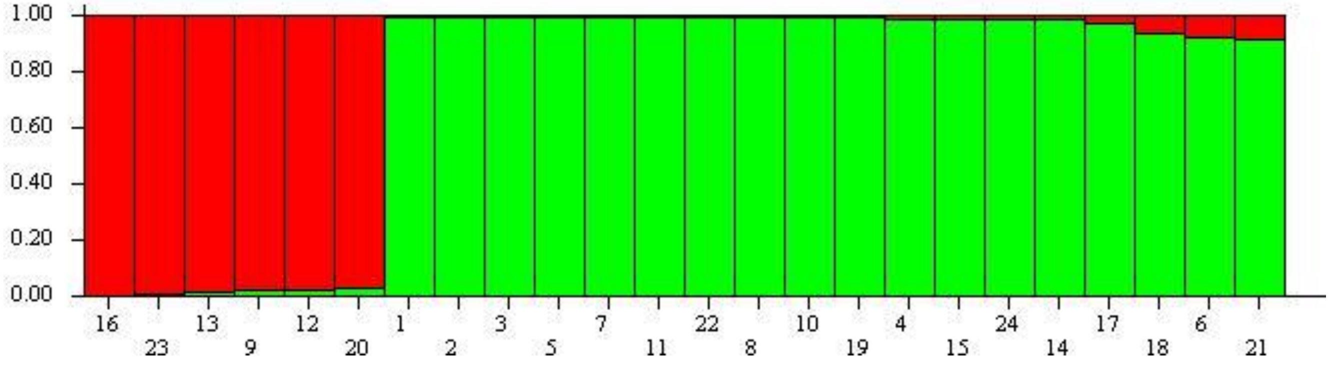

Figure S1b Structure analysis of *S. viminalis* when K = 2 clusters are assumed.

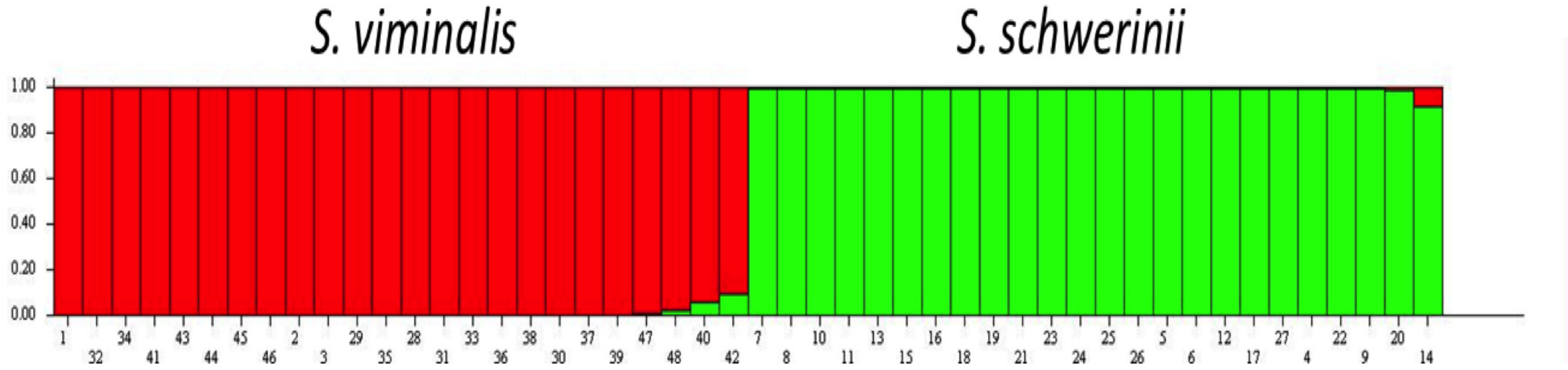

Figure S1c Structure analysis of *S. viminalis* and *S. schwerinii* when K = 2 clusters are assumed.
