# Supplementary material for: Polymorphism and Divergence in Two Willow Species, Salix viminalis L. and Salix schwerinii E. Wolf
Source: G3 (Bethesda). 2011 Oct 1;1(5):387–400. doi: 10.1534/g3.111.000539 (PMC3276148; doi:10.1534/g3.111.000539)
Supplement: Supporting Information [file supp_1.5.387_FigureS2.pdf]

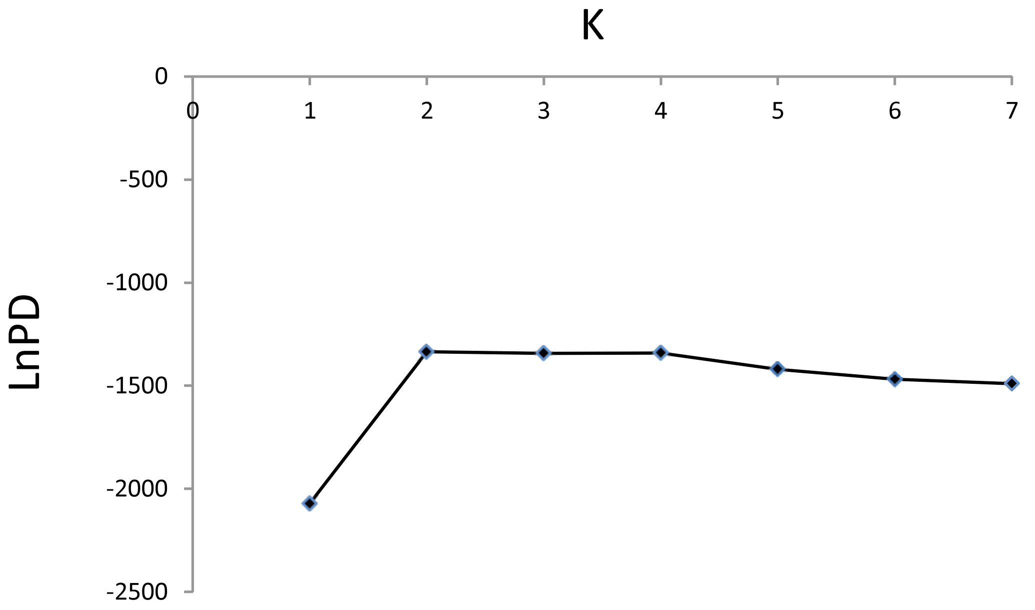

Figure S2a Estimated number of clusters (K) obtained with Structure when *S. schwerinii* and *S. viminalis* were run together. The mean LnPD is plotted over K (1-7).

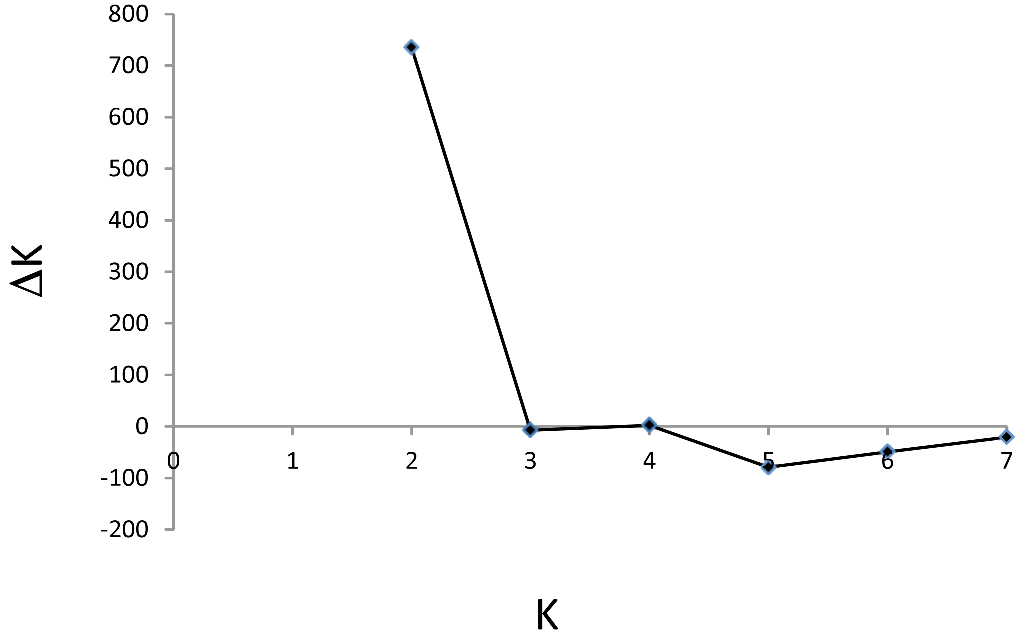

Figure S2b Estimated number of clusters (K) obtained with Structure when *S. schwerinii* and *S. viminalis* were run together. The mean ΔK is plotted over K (1-7).
