# Supplementary material for: Polymorphism and Divergence in Two Willow Species, Salix viminalis L. and Salix schwerinii E. Wolf
Source: G3 (Bethesda). 2011 Oct 1;1(5):387–400. doi: 10.1534/g3.111.000539 (PMC3276148; doi:10.1534/g3.111.000539)
Supplement: Supporting Information [file supp_1.5.387_TableS2.pdf]

Table S2 Primer sequences

Gene	Segment	Forward PCR primer 5´ to 3'	Reverse PCR primer 5´ to 3'
I-1	I-1A	TGATGGAAGACCTTTGTCTGG	GTCCAGCACAGCCTTTTTGT
	I-1B	CTTGGTTCGCAATCCAACACC	GGTTTGCTTGTTTCCACTGTC
I-53	I-53B*	CAGGTTTTGTGTGAGCTGGA	AGCAGGGCTCAGTGAATTGT
II-33	II-33A	AGGCTTTGCTTCTTTGGTCA	GCTGGACTTCCCAACATTAG
	II-33B	TAAGCATGTTTTCACCTGTGC	GCAGCATCCCTCAAATCAAT
II-36	II-36A	TGCTGTTTATTTGTTGTATGCAC	TCAGCAAATTGTAGCAAATGAA
	II-36B	TGAAGCAACAGTTCCAGGTG	GGGTGAGGAAAAGTCAACCA
III-4	III-4A	TTGTCTTCATGGTCGTGCAT	CAGCTTCCCATAAAGCGTTC
	III-4B	ACTGGAGCTGTTGTGCCTTT	CCACCAAGAGAGTGCCCTAC
III-24	III-24A	CCTGAATTGATGGCTGCATT	CACTTAGGCCCGGCAAATT
	III-24B	ATGATGGACGCAACAAAACT	TAGCCTTGGCCTCTTGAGAAG
IV-11	IV-11A	TGGTCACCACAAATTCGTCT	CATCTGCTGAGGACATGATTG
	IV-11B	CATGCCTCTCTCGGTTTCTC	AGTGCAAGCATGGGAAGTTG
IV-18	IV-18A	GTACAAGGCCGTGTTCCTGT	GCCATCGTCAGCTCAAACTC
	IV-18B	GAGTATCAGGAAATTGCAAGGC	CTGCTGAAGCTGCTCTAGCTG
V-18	V-18A	TGCTATCATGTTGCCCATGTG	TGGGCAAGTTTCCATCATAAG
	V-18B	GATTTCTGGAATAGTCGATTC	CAAGTTTCCATCATAAGTGC
V-20	V-20A	CCAAGGCTTTTATGGTGCAT	GAACTCCATCACCAAATTCCA
	V-20B	GCATGCAAGAAACAGGGTTTAC	AAGGATTGCATCCATTGCAG
VI-4	VI-4A	CAAGGATTGCTTGCCATGT	TGGCACAACAAATTCAGCTC
	VI-4B	GTGCTTCCACTTCGTGACTG	ATTGCAAATGGGACAGAACA
VII-1	VII-1A	GGCAGCAGAACCAAAGAAGA	TTCCTGGTGGCTAAAGTGGT
	VII-1B	CGATCCTGATGCTGCTGATA	GACAACAGTGGGAGCATCAA
VII-11	VII-11A	TTGGGAGAAGCTTTGCAACT	GGTGCAAAAGCATTTGTTGA
	VII-11B	ATTTTGGGCTTGGATTGTGA	TTGAGGTACATTGGCAGCAA
VIII-5	VIII-5A	TGGATTTCTGGACCAGGCTAC	GACAGGAGAATAGGAGCTTCG

	VIII-5B	TGCCATTCTACACCGAGTTG	AACCTGAAACCAGAGCACAG
VIII-14	VIII-14A	AAACTCCTGGAGAGGCAGG	CTGCACAAAGTCCATGTAGTC
	VIII-14B	TGCTTGGTGCTGACTTTTATG	ATGACCGTAACACCAGATCG
VIII-22	VIII-22B*	TCATGTCATTGAGGGTCGAA	TCCTGGTCTCCATCTGCATC
X-27	X-27A	TGTCAATGCCAAACCTTGAA	TTCAGTGGCTGGAACCTTCT
	X-27B	ACAGCCAAAACCAACACTCCG	ATCTCTCGTGGCACCTATGGA
XII-8	XII-8A	GGCATATTCTCTCCCTTGATG	GGGAGCCAGTTACCACCATA
	XII-8B	TGGTGACGATGAAGGCATTA	GGGTTCAGTCTTTGGAAGCA

*Only one segment per gene.
