# Supplementary material for: Polymorphism and Divergence in Two Willow Species, Salix viminalis L. and Salix schwerinii E. Wolf
Source: G3 (Bethesda). 2011 Oct 1;1(5):387–400. doi: 10.1534/g3.111.000539 (PMC3276148; doi:10.1534/g3.111.000539)
Supplement: Supporting Information [file supp_1.5.387_TableS3.pdf]

Table S3 P-values of obtaining multilocus summary statistic of the data under the individual species models. P-values are not corrected for multiple tests

Statistic	*S. schwerinii*			*S. viminalis*		
	Bottleneck	Growth	Neutral	Bottleneck	Growth	Neutral
Mean(S)	0,85	0,33	0,65	0,30	0,61	0,60
Mean(num_singleton)	0,46	0,08	0,20	0,08	0,13	0,13
Mean(πw)	0,76	0,23	0,47	0,24	0,48	0,46
Mean(TajD)	0,99	0,99	1	0,93	0,91	0,90
Mean(FuLiF*)	0,99	0,99	0,99	0,97	0,96	0,96
Mean(FuLiD*)	0,95	0,97	0,95	0,97	0,96	0,96
Mean(num_haplotypes)	0,76	0,23	0,70	0,30	0,58	0,55
Mean(DandVH)	0,68	0,16	0,52	0,11	0,21	0,15
Mean(WallsB)	0,61	0,71	0,50	0,94	0,91	0,91
Mean(WallsQ)	0,60	0,64	0,46	0,95	0,94	0,94
Var(S)	0,98	0,94	0,97	0,82	0,88	0,88
Var(num_singleton)	0,71	0,41	0,54	0,35	0,50	0,49
Var(πw)	0,60	0,25	0,38	0,32	0,46	0,44
Var(TajD)	0,88	0,96	0,88	0,98	0,98	0,97
Var(FuLiF*)	0,39	0,53	0,41	0,77	0,74	0,75

Var(FuLiD*)	0,13	0,20	0,14	0,66	0,64	0,66
Var(num_haplotypes)	0,92	0,92	0,95	0,91	0,90	0,90
Var(DandVH)	0,23	0,60	0,37	0,88	0,80	0,82
Var(WallsB)	0,50	0,76	0,48	0,69	0,57	0,55
Var(WallsQ)	0,46	0,70	0,42	0,60	0,48	0,45
