# Supplementary material for: Polymorphism and Divergence in Two Willow Species, Salix viminalis L. and Salix schwerinii E. Wolf
Source: G3 (Bethesda). 2011 Oct 1;1(5):387–400. doi: 10.1534/g3.111.000539 (PMC3276148; doi:10.1534/g3.111.000539)
Supplement: Supporting Information [file supp_1.5.387_TableS4.pdf]

TABLE S4 Number of outlier loci in the validation of the per species ABC models. Loci were considered outlier when two-sided P-value < 0.05. P values are not corrected for multiple tests.

Statistic	Bottleneck	S. schwerinii	Neutral	Bottleneck	S. viminalis	Neutral
		Growth			Growth	
Segregating sites	2	1	2	1	1	1
Singletons	0	0	0	0	0	0
Wattersons theta	2	1	2	1	1	1
Tajimas D	4	4	4	2	2	2
Fu & Li F*	3	3	3	2	2	2
Fu & Li D*	1	1	1	2	2	2
Number of haplotypes	1	1	1	2	2	3
