# Supplementary material for: Polymorphism and Divergence in Two Willow Species, Salix viminalis L. and Salix schwerinii E. Wolf
Source: G3 (Bethesda). 2011 Oct 1;1(5):387–400. doi: 10.1534/g3.111.000539 (PMC3276148; doi:10.1534/g3.111.000539)
Supplement: Supporting Information [file supp_1.5.387_TableS5.pdf]

Table S5 Outlier loci in the validation of ABC models. Number of statistics for which the loci were considered to be outlier at α=0.05 level, without correction for multiple tests.

Locus	Bottleneck	S. schwerinii Growth	Neutral	Bottleneck	S. viminalis Growth	Neutral	Split	Split without migration	No split
I-1	1	1	1	3	3	3	3	3	6
I-53b									3
II-33									3
II-36				2	2	2			4
III-4c							3	2	3
IV-11									3
IV-18	5	4	5					1	7
V-18b									2
VI-4	2	2	2	3	3	3			5
VII-11	3	3	3			1	3	1	2
VII-1				2	2	2			3
VIII-14								1	2
VIII-5									2
X-27	2	2	2						4
