# Supplementary material for: Polymorphism and Divergence in Two Willow Species, Salix viminalis L. and Salix schwerinii E. Wolf
Source: G3 (Bethesda). 2011 Oct 1;1(5):387–400. doi: 10.1534/g3.111.000539 (PMC3276148; doi:10.1534/g3.111.000539)
Supplement: Supporting Information [file supp_1.5.387_TableS6.pdf]

Table S6 Number of outlier loci in the validation of the population split ABC models. Loci were considered outlier when two-sided P-value < 0.05. P values are not corrected for multiple tests.

Statistic	Split	Split without migration	No split
Segregating sites $_{S. schwerinii}$			
Singletons $_{S. schwerinii}$			
Wattersons theta $_{S. schwerinii}$			
Tajimas D $_{S. schwerinii}$			4
Fu & Li F* $_{S. schwerinii}$			3
Fu & Li D* $_{S. schwerinii}$		1	1
Number of haplotypes $_{S. schwerinii}$	1	1	
Segregating sites $_{S. viminalis}$			
Singleton $_{S. viminalis}$			
Wattersons theta $_{S. viminalis}$			
Tajimas D $_{S. viminalis}$	1	1	4
Fu & Li F* $_{S. viminalis}$	1	1	3
Fu & Li D* $_{S. viminalis}$	1	1	2
Number of haplotypes $_{S. viminalis}$			
Segregating sites $_{S. schwerinii + S. viminalis}$			
Singletons $_{S. schwerinii + S. viminalis}$	2		
Wattersons theta $_{S. schwerinii + S. viminalis}$			
Tajimas D $_{S. schwerinii + S. viminalis}$	1	1	2
Fu & Li F* $_{S. schwerinii + S. viminalis}$	1	1	1
Fu& Li D* $_{S. schwerinii + S. viminalis}$			1
Number of haplotypes $_{S. schwerinii + S. viminalis}$	1		
Fst		1	14
Number of shared polymorphisms			
Number of fixed polymorphisms			14
Number of private polymorphisms $_{S. schwerinii}$			
Number of private polymorphisms $_{S. viminalis}$			
