# Supplementary material for: Polymorphism and Divergence in Two Willow Species, Salix viminalis L. and Salix schwerinii E. Wolf
Source: G3 (Bethesda). 2011 Oct 1;1(5):387–400. doi: 10.1534/g3.111.000539 (PMC3276148; doi:10.1534/g3.111.000539)
Supplement: Supporting Information [file supp_1.5.387_TableS7.pdf]

**Table S7 P-values of obtaining multilocus summary statistic of the data under three different models. P values are not corrected for multiple tests.**

| Stat | Split | Split no migration | No split |
|---|---|---|---|
| Mean($S_{S.\ schwerinii}$) | 0.72 | 0.43 | 0.87 |
| Mean($singleton_{S.\ schwerinii}$) | 0.71 | 0.47 | 0.86 |
| Mean($\pi w_{S.\ schwerinii}$) | 0.67 | 0.36 | 0.87 |
| Mean($TajD_{S.\ schwerinii}$) | 0.85 | 0.80 | 0.93 |
| Mean($FuLiF*_{S.\ schwerinii}$) | 0.72 | 0.57 | 0.88 |
| Mean($FuLiD*_{S.\ schwerinii}$) | 0.61 | 0.42 | 0.74 |
| Mean($num\_haplo_{S.\ schwerinii}$) | 0.72 | 0.40 | 0.89 |
| Mean($DandVH_{S.\ schwerinii}$) | 0.64 | 0.31 | 0.88 |
| Mean($WallsB_{S.\ schwerinii}$) | 0.38 | 0.23 | 0.52 |
| Mean($WallsQ_{S.\ schwerinii}$) | 0.39 | 0.22 | 0.54 |
| Mean($S_{S.\ viminalis}$) | 0.68 | 0.52 | 0.87 |
| Mean($singleton_{S.\ viminalis}$) | 0.63 | 0.57 | 0.85 |
| Mean($\pi w_{S.\ viminalis}$) | 0.65 | 0.48 | 0.86 |
| Mean($TajD_{S.\ viminalis}$) | 0.46 | 0.25 | 0.79 |
| Mean($FuLiF*_{S.\ viminalis}$) | 0.59 | 0.38 | 0.80 |
| Mean($FuLiD*_{S.\ viminalis}$) | 0.64 | 0.46 | 0.75 |
| Mean($num\_haplo_{S.\ viminalis}$) | 0.59 | 0.49 | 0.87 |
| Mean($DandVH_{S.\ viminalis}$) | 0.45 | 0.32 | 0.86 |
| Mean($WallsB_{S.\ viminalis}$) | 0.66 | 0.43 | 0.81 |
| Mean($WallsQ_{S.\ viminalis}$) | 0.69 | 0.45 | 0.85 |
| Mean($S_{combined}$) | 0.61 | 0.44 | 0.88 |
| Mean($singleton_{combined}$) | 0.73 | 0.53 | 0.87 |
| Mean($\pi w_{combined}$) | 0.55 | 0.35 | 0.88 |
| Mean($TajD_{combined}$) | 0.56 | 0.68 | 0.92 |
| Mean($FuLiF*_{combined}$) | 0.52 | 0.56 | 0.89 |
| Mean($FuLiD*_{combined}$) | 0.49 | 0.47 | 0.73 |
| Mean($num\_haplo_{combined}$) | 0.68 | 0.42 | 0.91 |
| Mean($DandVH_{combined}$) | 0.52 | 0.29 | 0.90 |

| | | | |
|---|---|---|---|
| Mean(WallsB$_{combined}$) | 0.23 | 0.18 | 0.54 |
| Mean(WallsQ$_{combined}$) | 0.24 | 0.19 | 0.55 |
| Mean(Fst) | 0.44 | 0.57 | 1.00 |
| Mean(shared) | 0.68 | 0.49 | 0.81 |
| Mean(fixed) | 0.30 | 0.26 | 1.00 |
| Mean(private$_{S. schwerinii}$) | 0.76 | 0.46 | 0.90 |
| Mean(private$_{S. viminalis}$) | 0.70 | 0.58 | 0.90 |
| Var(S$_{S. schwerinii}$) | 0.86 | 0.78 | 0.89 |
| Var(singleton$_{S. schwerinii}$) | 0.73 | 0.58 | 0.87 |
| Var(πw$_{S. schwerinii}$) | 0.58 | 0.32 | 0.87 |
| Var(TajD$_{S. schwerinii}$) | 0.77 | 0.86 | 0.81 |
| Var(FuLiF*$_{S. schwerinii}$) | 0.44 | 0.57 | 0.52 |
| Var(FuLiD*$_{S. schwerinii}$) | 0.24 | 0.35 | 0.46 |
| Var(num_haplo$_{S. schwerinii}$) | 0.97 | 0.93 | 0.91 |
| Var(DandVH$_{S. schwerinii}$) | 0.26 | 0.52 | 0.45 |
| Var(WallsB$_{S. schwerinii}$) | 0.34 | 0.40 | 0.69 |
| Var(WallsQ$_{S. schwerinii}$) | 0.34 | 0.41 | 0.69 |
| Var(S$_{S. viminalis}$) | 0.78 | 0.65 | 0.88 |
| Var(singleton$_{S. viminalis}$) | 0.70 | 0.64 | 0.86 |
| Var(πw$_{S. viminalis}$) | 0.60 | 0.42 | 0.86 |
| Var(TajD$_{S. viminalis}$) | 0.91 | 0.87 | 0.95 |
| Var(FuLiF*$_{S. viminalis}$) | 0.74 | 0.76 | 0.77 |
| Var(FuLiD*$_{S. viminalis}$) | 0.71 | 0.76 | 0.66 |
| Var(num_haplo$_{S. viminalis}$) | 0.66 | 0.62 | 0.90 |
| Var(DandVH$_{S. viminalis}$) | 0.54 | 0.65 | 0.82 |
| Var(WallsB$_{S. viminalis}$) | 0.48 | 0.36 | 0.74 |
| Var(WallsQ$_{S. viminalis}$) | 0.43 | 0.34 | 0.74 |
| Var(S$_{combined}$) | 0.90 | 0.87 | 0.89 |
| Var(singleton$_{combined}$) | 0.93 | 0.89 | 0.89 |
| Var(πw$_{combined}$) | 0.39 | 0.23 | 0.87 |
| Var(TajD$_{combined}$) | 0.85 | 0.91 | 0.72 |

| | | | |
|---|---|---|---|
| Var(FuLiF*$_{combined}$) | 0.85 | 0.88 | 0.62 |
| Var(FuLiD*$_{combined}$) | 0.71 | 0.74 | 0.52 |
| Var(num_haplo$_{combined}$) | 0.98 | 0.95 | 0.92 |
| Var(DandVH$_{combined}$) | 0.60 | 0.73 | 0.42 |
| Var(WallsB$_{combined}$) | 0.31 | 0.40 | 0.69 |
| Var(WallsQ$_{combined}$) | 0.37 | 0.46 | 0.70 |
| Var(Fst) | 0.75 | 0.80 | 1.00 |
| Var(shared) | 0.76 | 0.61 | 0.86 |
| Var(fixed) | 0.28 | 0.24 | 1.00 |
| Var(private$_{S.\ schwerinii}$) | 0.89 | 0.81 | 0.90 |
| Var(private$_{S.\ viminalis}$) | 0.77 | 0.69 | 0.90 |
